# Comparing the impact and mechanistic pathways of micro-environmental interventions targeting healthier vs. more environmentally sustainable food options: an overview of reviews

**DOI:** 10.1186/s12916-025-04381-8

**Published:** 2025-10-24

**Authors:** Cinja Jostock, Elizabeth Biggs, Ethan Knight, Madison Luick, Nia Roberts, Gareth J. Hollands, Rachel Pechey

**Affiliations:** 1 Nuffield Department of Primary Care Health Sciences, University of Oxford, Radcliffe Primary Care Building, Radcliffe Observatory Quarter, Woodstock Rd, Oxford, OX2 6GG UK; 2 Bodleian Health Care Libraries, University of Oxford, Oxford, UK; 3 EPPI Centre, UCL Social Research Institute, University College London, London, UK

**Keywords:** Micro-environment, Interventions, Choice architecture, Healthy food, Sustainable food, Overview of reviews

## Abstract

**Background:**

The potential for interventions that target food environments to influence dietary behaviour has been explored for both healthier and more environmentally sustainable diets, but the extent to which health-focused and sustainability-focused interventions can inform each other is unclear. This overview of reviews compares the characteristics and effectiveness of micro-environmental interventions aimed at health versus sustainability and explores their mediators and moderators.

**Methods:**

We searched 10 databases for systematic reviews including randomised controlled trials of micro-environmental interventions targeting healthier or more sustainable food choices. We conducted forwards and backwards citation tracking of included reviews. Review quality was assessed using AMSTAR2. We narratively synthesised results, categorising interventions using the TIPPME typology of micro-environmental interventions.

**Results:**

We screened 4154 records and included 31 reviews, of which 26 targeted health and 5 sustainability. Of 228 interventions, 31 (13.6%) targeted sustainability, 194 (85.1%) targeted health, and 3 (1.3%) targeted both. There was little overlap between the intervention types investigated by health and sustainability interventions. Size and position interventions were most common for health interventions, whilst information and presentation interventions were the most frequent sustainability interventions. Default, size, and menu positioning interventions appear particularly promising for both health and sustainability benefits, albeit with limited evidence for the latter in particular. Evidence of effect modifiers was scarce. Almost all reviews had a “critically low” or “low” confidence rating based on the AMSTAR2, limiting confidence in their estimates of intervention effectiveness.

**Conclusions:**

There is more evidence for health-focused interventions than sustainability-focused interventions. Size and position interventions seem most promising, but evidence for sustainability is scarce. There is currently no evidence of differential responding to health vs. sustainability interventions, although we were unable to comprehensively assess this. More comparable evidence, and evidence on underlying mechanisms, is needed, prioritising the most effective interventions.

**Supplementary Information:**

The online version contains supplementary material available at 10.1186/s12916-025-04381-8.

## Background

The food we consume affects both our health and the environment, with current consumption trends negatively impacting both population and planetary health [1]. We can potentially tackle both issues simultaneously, as more sustainable foods also tend to be healthier: More sustainable diets often involve greater quantities of plant-based foods, including fruits and vegetables, and lower quantities of animal-sourced foods [2–4]. This correlates with the Mediterranean diet, characterised by a low red and processed meat intake and high intake of fruit and vegetables, which has been linked to better long-term health outcomes [5]. One element of particular concern from a sustainability perspective is meat, production of which entails a considerably higher environmental impact than that of plant-based foods [6], whilst consumption of red and processed meat has been linked to adverse health outcomes [7, 8]. However, considering that the demand for meat and dairy products is rising globally [9], a shift towards increasingly plant-based diets may be challenging [10].

Micro-environmental interventions, which modify the choice environment to influence behaviour [11], may help encourage behavioural shifts towards both healthier and more sustainable foods, but research has tended to focus on the former [12–16]. Given the high environmental impact of the food system [17] contributing to anthropogenic climate change [18], it is important to build and maintain equivalent evidence bases for healthier and for more environmentally sustainable diets (hereafter, for better readability, we use the word “sustainable” to refer to environmental sustainability). This could be expedited if the evidence base for healthier dietary interventions can be used to draw parallels for sustainable diets, such that one combined evidence base could inform recommendations across both healthier and sustainable food targets. However, whilst correlated, healthier foods are not always more sustainable and vice versa, and the most impactful products to target for health do not always correspond with those for sustainability [2]. The extent to which these evidence bases are complementary has not been assessed.

Whilst healthier and sustainable food targets show some clear overlap, if intervention effectiveness is moderated by key differences between commonly targeted healthier and more sustainable foods, interventions that prove effective for healthier foods may not be equally effective for more sustainable foods. For example, the salience (i.e. noticeability) of interventions, and motivation for selecting food options (including preferences) may differ between targeted healthier and more sustainable foods. Interventions manipulating meat versus vegetarian options (compared to meal healthiness) may be more conspicuous, as meal presentations and descriptions often centre on meat. This may reflect existing strong social and cultural norms regarding meat consumption [19], and learnt preferences that play a key role in determining food selection and consumption [20], and may counteract dietary interventions. Indeed, meat is regarded as “normal, nice, necessary and natural” [21], with readiness to eat less meat being low [22, 23]. In contrast, most people aspire to eat healthily [24, 25]. Moreover, the healthiness of foods is perceived as more important than sustainability of foods when making food decisions [26–28], so that interventions aimed at healthier food choices may have the advantage of a more motivated audience. This may be exacerbated given public understanding of the links between food and health is generally high [24, 25], whereas the impact of food choices on sustainability is less well established [23]. Acquiring new knowledge of the impact of selecting particular foods may be a necessary prerequisite to—or moderator of—intervention effectiveness [29]. However, the impact of knowledge may be limited if willingness remains low. In addition, there are gender discrepancies for both healthier and more sustainable eating behaviours: women are more open to meat reduction than men [22, 23] and are more likely to attempt adhering to a healthier diet [30]. However, it is unclear if these gender discrepancies are of the same magnitude for healthier versus more sustainable diets. Systematic investigation of such potential moderating or mediating factors could identify whether or not equivalency in intervention effectiveness when targeting healthier vs. more sustainable food might be expected.

However, to the best of our knowledge, past overviews of reviews have investigated interventions that encourage either healthier or environmentally sustainable diets in isolation (e.g. [31–33]), and/or focused on one particular group characteristic (e.g. lower income) [34] or context (e.g. schools) [35–38]. By contrast, this review aims to provide the evidence base to robustly compare findings between interventions targeting health and environmental sustainability, for which we need to look across a range of both interventions and settings. In addition, we aim to identify potential moderating and mediating factors. A systematic scoping review of choice architecture interventions generally found that out of the reviewed studies, around three quarters focused on evaluating the intervention’s applicability in a particular setting, whilst only approximately one quarter primarily aimed to investigate moderators or underlying processes of the interventions [39]. Similarly, evidence surrounding potential moderators that affect the success of interventions aiming to promote more sustainable dietary choices is scarce [12].

In this overview of reviews, we aim to compare the effectiveness of micro-environmental interventions that aim to promote healthier versus more environmentally sustainable dietary choices. We focus on systematic reviews of interventions that target the selection, purchase, or consumption of (a) healthier vs. less healthy foods and (b) more vs. less sustainable foods. We also explore any potential mediators and moderators that may influence the effectiveness of interventions as well as any cross-cultural factors. To the best of our knowledge, this is the first overview of reviews that compares the characteristics and effectiveness of micro-environmental interventions targeting healthier food choices with those that target more sustainable food choices.

## Methods

The review protocol was registered with PROSPERO [CRD42022382577]. We followed the PRIOR statement for reporting overviews of reviews [40].

### Eligibility criteria

Eligibility criteria following the PICOS framework [41] are outlined in Table 1.

**Table 1:** Eligibility criteria

Population	No restrictions
Intervention	Micro-environmental interventions defined according to the TIPPME framework, which divides micro-environmental interventions into six different types of interventions [11]. We added more stringent criteria for information interventions to limit this to simpler forms of information provision that may be less likely to require cognitive processing.*
Comparison/Comparator	Intervention vs. control (no intervention, default, or the same intervention implemented to a different extent)
Outcomes	Actual or intended** selection, purchase or consumption of (i) healthier, (ii) less healthy, (iii) more sustainable, or (iv) less sustainable food options***, including non-alcoholic drinks. Both actual and intended selection, purchase, and consumption were considered
Study type	Systematic reviews that exclusively include RCTs (between-subjects, within-subjects (crossover), and cluster RCTs) or where the results for RCTs are reported separately from other study types such as observational studies. A review is defined as “systematic” if the authors define it as such, and it clearly reports the search strategy, study identification process, inclusion/exclusion criteria and process, and risk of bias assessment

*Further information on our definition of information intervention is given in Appendix 2

**Intended/hypothetical outcomes are those where participants did not actually receive or consume studied products

***Healthier: Reviews that identify their target as healthier (including low(er) energy, sugar, fat or salt; high(er) fibre) food options; or targeting fruit or vegetables. Less healthy: Reviews that identify their target as less healthy (including high(er) energy, sugar, fat or salt; low(er) fibre) food options; or targeting food categories that can be classed as discretionary foods following the Food Standards Scotland [42] classification (i.e. confectionery, sweet biscuits, crisps, savoury snacks, cakes, sweet pastries and puddings). More sustainable: Reviews that identify their target as more sustainable/lower environmental impact; or targeting plant-based, vegan or vegetarian options. Less sustainable: Reviews that identify their target as less sustainable/higher environmental impact; or targeting meat

Interventions including additional components that were not micro-environmental according to the TIPPME framework or that were not aimed at encouraging healthier or more sustainable food choices were only included if this component was also present in the comparator. We excluded reviews where results for micro-environmental interventions or outcomes of interest could not be separated from other interventions or outcomes.

### Search strategy

We searched 10 databases from inception to 10th January 2023 (Appendix 1). A limit was applied to focus on systematic reviews, no date or language limits were applied. We conducted forward and backward searches of included reviews on 23rd October 2023, using the citation chaser web app [43]. We excluded any reviews published after the main search date.

### Review selection

For the main search, two reviewers screened all title-abstract records and full-text reports independently using Covidence [44], with conflicts resolved by a third reviewer when needed. References identified through forward and backward citation tracking were screened by one reviewer, with a random 20% subsample screened by a second reviewer.

### Data extraction

For the main search, two reviewers independently extracted key data (outcomes, results) in duplicate using Covidence [44] and created a list of all eligible studies in Microsoft Excel to avoid double-counting [45, 46]. Conflicts were resolved by a third reviewer. Following established guidance [47], we extracted review data on first author, title, country, funding source, aim, review type, topic, number of databases searched and date range, date of last search update, eligibility criteria, method of synthesis, method of quality assessment, limitations, and review author comments regarding specific studies; and of included relevant studies: number of studies, publication date range per outcome of interest, study design, country, participants, settings, outcomes, outcome measurements, findings, identified mediators, moderators, and cross-cultural factors, and quality rating. We generally extracted information at review level; however, if insufficient descriptions were provided by reviews, we double checked eligibility by referring to primary studies.

For the citation tracking, one reviewer extracted data for all reviews, with a second reviewer extracting key data for a random 20% subsample. Conflicts were resolved by discussion, with a third reviewer arbitrating if necessary.

We coded interventions according to the six TIPPME “intervention type” categories [11] (Table 2), coding every relevant intervention arm separately when a study tested several interventions. We expanded the scope to online and laboratory studies, added a multi-component category, and further specified the definition for information interventions as we aimed to only include information interventions that provide limited information and thus limit the need for cognitive processing [48]. For example, we included short social norms messages but excluded more information-heavy or interactive interventions such as taste tests or handing out flyers. Examples of interventions that we included or excluded are in Appendix 3.

**Table 2:** TIPPME framework [11]

Intervention type	Definition
*Availability*	Interventions that add or remove (some or all) products or objects to increase, decrease, or change their range, variety or number
*Position*	Interventions that alter the position, proximity, or accessibility of products or objects
*Functionality*	Interventions that alter functionality or design of products or objects to change how they work, or guide, or constrain how people use or physically interact with them
*Presentation*	Interventions that alter visual, tactile, auditory, or olfactory properties of products, objects, or stimuli
*Size*	Interventions that alter the size or shape of products or objects
*Information*	Interventions that convey simple information about a product or object or its use through adding, removing, or changing words, symbols, numbers, or pictures. For this review, we defined simple information as including the use of summary labels (e.g. Nutri-Score labels) but not nutrient-specific labels (e.g. kcal labels) [49–52]
*Multicomponent*	Any combination of the above categories

Definitions for availability, position, functionality, presentation, and size interventions are fully adapted from the TIPPME framework [11]. The TIPPME definition of information interventions has been further specified to better align it with the purpose of this study: “Information interventions that only make use of a message that is expected to be visually salient (i.e. clearly seen on the typical path through the environment) and target one specific behaviour change”

If interventions reported several relevant outcomes (e.g. selection and consumption), we included all outcomes. When reviews reported several relevant measures for the same outcome, the most comprehensive (e.g. total calories over fat content of food), and if not discernible, the most relevant measure to the study question was chosen. This was decided between the two reviewers with conflicts resolved by a third reviewer if necessary. We assigned interventions a positive effect (+) if they reported a positive outcome in terms of healthiness/sustainability, a 0 if no effect was found, and (−) for a negative effect.

### Risk of bias

For the main search, two reviewers independently assessed the risk of bias of included reviews using the AMSTAR2 tool [53]. Reviews identified through citation tracking were assessed by one reviewer, with a random 20% subsample assessed by a second reviewer. We resolved any conflicts through discussion. We rated the overall confidence of reviews ranging from “critically low” to “high” [53]. We made a minor amendment to the recommended critical items for the rating: we removed item 7 (“listing all excluded studies with exclusion reasons”) as this was deemed less important for our purposes. Thus, we consider questions 2, 4, 9 (for RCTs), 11, 13, and 15 critical domains. Additionally, we value “partial yes” as a “yes” since no recommendation regarding this is made by the AMSTAR2 authors, and the criteria for “yes” are fairly strict.

### Data synthesis

We conducted a narrative synthesis [54] of included reviews. We tabulated and described each review and grouped them by intervention type using the TIPPME framework [11] (Table 2) and by our outcome categories (hypothetical, selection/purchase, consumption) to explore patterns. Where a large number of interventions was included for a single TIPPME category, we sub-categorised these. We critically reflected on the risk of bias of included reviews and how this may impact the validity of our results.

## Results

**Fig. 1:**
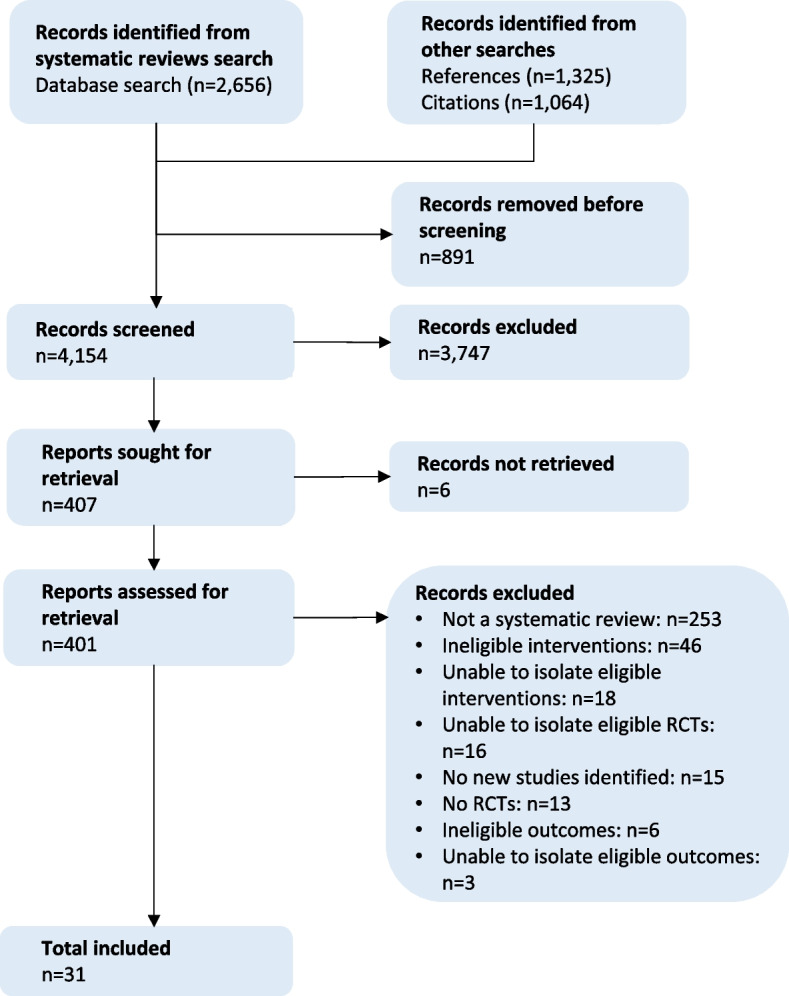
PRIOR flowchart

We screened 4154 records and included 31 reviews (Fig. 1). We included each primary study only once, except for three interventions that were included in both health- and sustainability-focused reviews due to differing outcomes of interest (overall/salad vs. meat consumption). None of the reviews exclusively included studies that were eligible; we thus extracted subsets of eligible studies (Table 3).

**Table 3:** Characteristics of included systematic reviews

Review ID	Aim	Population	Eligible N*	Outcomes			Intervention types							Quality rating of primary studies
				Hypothetical	Selection/Purchase	Consumption	Availability	Position	Functionality	Presentation	Size	Information	Multi-component	
Al-Khudairy_2019 [55]	H	NHS/healthcare staff, developed countries	1		X						X			Low risk for 3/7 assessed domains, unclear for 4
An_2021 [56]	H	NR	3	X	X							X		6/8 (*n* = 2); 5/8 (*n* = 1)
Atanasova_2022 [57]	H	Adults and children	3	X	X	X	X	X				X		Low risk (*n* = 3)
Bianchi_2018 [58]	S	All, excl. those with conditions requiring meat intake	9 (13)	X	X	X		X		X	X	X		Strong (*n* = 1); medium (*n* = 4); low (*n* = 4)
Blackford_2021 [13]	S	NR	1 (3)		X							X		Moderate
Cameron_2016 [59]	H	NR [interventions in supermarkets, grocery or online stores]	1	X								X		Moderate
Chu_2021 [60]	H	All ages; no patients or those with restrictive diets	2			X				X	X			81%
Funderburk_2020 [61]	H	Human subjects	1			X							X	Not reported, at least 70
Golding_2022 [62]	H	In-store supermarket customers	3		X							X		Moderate
Gupta_2021 [63]	H	Any human population	1	X								X		Moderate
Gynell_2022 [64]	H	Generic human population (excl. those with overweight/obesity or specific illnesses)	12 (18)	X	X	X		X		X		X		5 stars (*n* = 2); 4 stars (*n* = 4); 3 stars (*n* = 5); 2 stars (*n* = 1)
Harbers_2020 [67]	H	Adults	2		X			X				X		Moderate
Hartmann-Boyce_2018 [68]	H	All	1 (2)	X								X		High risk
Hersey_2013 [51]	H	NR	2	X		X						X		7/10 (*n* = 1); 6/10 (*n* = 1)
Hollands_2015 [65]	H	Adults and children	71 (107)		X	X			X	X	X			Consumption: high risk (*n* = 5), unclear (*n* = 52) Selection: unclear (*n* = 7), high (*n* = 2)
Hollands_2019 [66]	H	Adults and children (excl. if one person selected and fed foods directly to another person)	16 (19)		X	X	X	X						High risk (*n* = 1); some concerns (*n* = 12); low risk (*n* = 3)
Kraak_2015 [69]	H	Children (2–12 years)	2 (3)	X						X				Medium causal inference validity and ecological validity, no use of conceptual model/analytical framework (*n* = 2)
Laiou_2021 [70]	H	All populations	3 (5)		X	X		X	X	X				6 domains unclear (*n* = 2); 3 high risk, 2 unclear, 1 low (*n* = 1)
Ljusic_2022 [71]	H	NR	1	X								X		High risk
Marcano-Olivier_2020 [72]	H	Typical school populations	1 (2)		X	X	X	X						Moderate
Mathur_2021 [73]	S	Any human population	2 (3)	X						X				Low risk (*n* = 1); unclear (*n* = 1)
Meier_2022 [74]	S	Individuals/households consuming animal protein/substitutes	5		X			X			X			Low risk (*n* = 2); moderate (*n* = 3)
Metcalfe_2020 [75]	H	School children (Kindergarten to grade 12)	4		X	X				X			X	Moderate
Osei-Assibey_2012 [76]	H	Children under age 9 (excl. if only focused on childhood obesity or on participants with overweight/obesity)	1		X							X		Not reported
Richardson_2022 [77]	H	Employees/visitors to hospital food environments	1		X		X							Quality/risk of bias targets met
Shaw_2020 [78]	H	Adults, high-income countries	1		X			X						High risk
Slapø_2021 [79]	H	All populations within grocery stores	1	X**								X		6/7 domains low risk, one unclear
Stiles_2022 [80]	S	NR [interventions had to be in commercial food services]	8 (10)		X							X		1Y, 2NR, 2 N (*n* = 3); 3Y, 2NR (*n* = 1); 2Y, 2NR, 1 N (*n* = 3); 2Y, 3NR (*n* = 1)
Vargas-Alvarez_2021 [81]	H	Healthy adults/children, or those with condition not influencing daily activities	8 (9)	X	X	X					X			8 domains low risk (*n* = 1); 4 low, 2 N/A, 1 unclear, 1 high (*n* = 1); 4 low, 2 N/A, 2 high (*n* = 1); 5 low, 2 N/A, 1 high (*n* = 1); 5 low, 2 unclear, 1 high (*n* = 1); 6 low, 2 unclear (*n* = 1); 7 unclear, 1 high (*n* = 1); 6 low, 1 unclear, 1 high (*n* = 1)
Whatnall_2020 [82]	H	University staff/students [or interventions in university settings]	1		X							X		Positive
Wilson_2016 [83]	H	Adults	2 (3)		X			X						Good (*n* = 1); average (*n* = 1)

*H* Health, *S *Sustainability, *NR *not reported

*Refers to number of studies included in this overview of reviews, not the systematic review overall; number of study arms (i.e. interventions) included in brackets

** We class this outcome as hypothetical; however, participants had a 1 in 3 chance of actually having to pay for and receiving products they selected

Most reviews were aimed at health (*n* = 26) [51, 55–57, 59–72, 75–79, 81–83], with five aimed at sustainability [13, 58, 73, 74, 80] (Table 3). Reviews were published between 2012 [76] and 2022 [57, 62, 64, 71, 74, 77, 80]. Most reviews aimed at sustainability were published in 2021 or 2022 [13, 73, 74, 80], reflecting increasing interest in sustainable dietary behaviours. Only five reviews used meta-analysis to summarise studies [65, 66, 73, 79, 81]; the remainder (*n* = 26) summarised results narratively [13, 51, 55–64, 67–72, 74–78, 80, 82, 83], using for example qualitative comparative analysis [58, 68].

Most reviews were conducted in the UK (*n* = 12) [13, 55, 57, 60, 62, 65, 66, 68, 72, 76–78], followed by Australia (*n* = 5) [59, 63, 64, 82, 83], and the USA (*n* = 5) [51, 61, 69, 73, 75], Norway (*n* = 2) [71, 79], and the Netherlands (*n* = 1) [67]. The remaining reviews were conducted by authors situated in multiple high-income countries (*n* = 4) [58, 70, 80, 81] or by authors affiliated with universities from high- and upper-middle income countries (*n* = 2) [56, 74]. Similarly, for individual studies, in 25 out of 28 reviews that reported sufficient information [13, 51, 55–70, 72–79, 81, 82], all extracted studies were conducted in high-income countries [13, 51, 55–64, 66–69, 72–79, 82]. Common settings included schools or universities, online settings, food stores, restaurants, laboratories, and worksites.

Of the 228 included interventions, 31 (13.6%) were aimed at sustainability, 194 (85.1%) were aimed at health, and we counted 3 (1.3%) towards health and sustainability. The most popular intervention types for health were size (*n* = 116 (60%)) and positioning (*n* = 36 (19%)) interventions, but for sustainability, information (*n* = 17 (55%)), and presentation (*n* = 7 (23%)) interventions were most common. Functionality (health: *n* = 3; sustainability: *n* = 0), multicomponent (health: *n* = 4; sustainability: *n* = 0), and availability (health: *n* = 8; sustainability: *n* = 0) interventions were less commonly tested. Most interventions aimed at health were from a Cochrane review of size interventions (*n* = 107; 55%) [65].

## Effectiveness of different types of micro-environmental interventions

### Availability

**Fig. 2:**
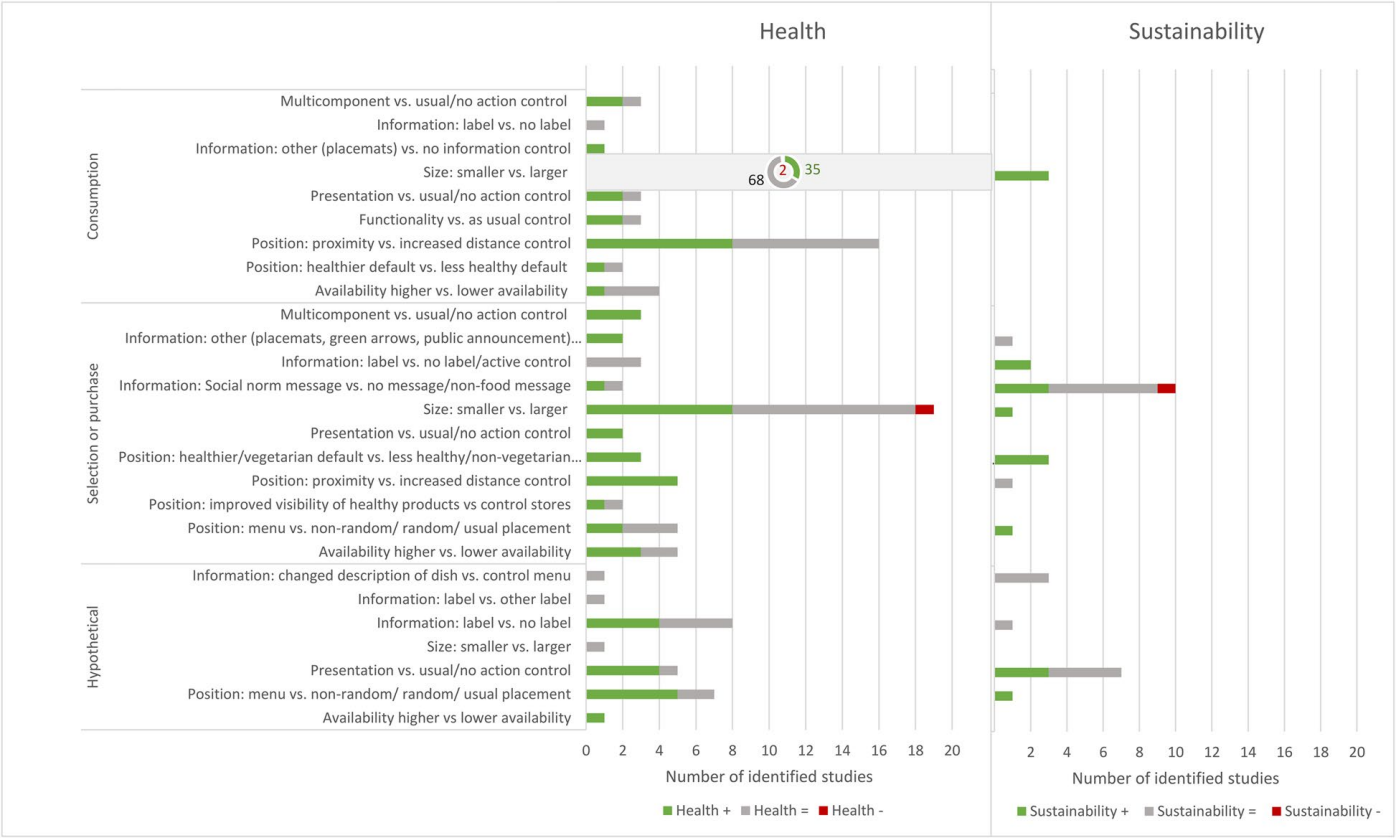
Summary of results. “’+’: Positive effect; ‘=’: No effect; ‘-‘: negative effect; N.B. Size for Health not plotted (105 studies identified)“

Five reviews [57, 66, 72, 76, 77] included eight availability interventions (Table 4) (Fig. 2).

**Table 4:** Summary of results by TIPPME [11] intervention type and comparator

Intervention type and comparator	Health					Sustainability				
	**Effect**			***N***	**Reviews**	**Effect**			***N***	**Reviews**
	** + **	** = **	** − **			** + **	** = **	** − **		
Hypothetical outcomes (***n*** = 36)										
Availability higher vs lower availability	1	/	/	1	Atanasova_2022	/	/	/	/	/
Position: menu vs. non-random/random/usual placement	5	2	/	7	Gynell_2022	1	/	/	1	Bianchi_2018
Presentation vs. usual/no action control	4	1	/	5	Gynell_2022; Kraak_2015	3	4	/	7	Bianchi_2018; Mathur_2021
Size: smaller vs. larger	/	1	/	1	Vargas-Alvarez_2021	/	/	/	/	/
Information: label vs. no label	4	4	/	8	An_2021; Cameron_2016; Gupta_2021; Hartmann-Boyce_2018; Hersey_2013; Ljusic_2022; Slapø_2021	/	1	/	1	Bianchi_2018
Information: label vs. other label	/	1	/	1	An_2021	/	/	/	/	/
Information: changed description of dish vs. control menu	/	1	/	1	Gynell_2022	/	3	/	3	Bianchi_2018
Selection or Purchase (***n*** = 70)										
Availability higher vs. lower availability	3	2	/	5	Hollands_2019; Marcano-Olivier_2020; Osei-Assibey_2012; Richardson_2022	/	/	/	/	/
Position: menu vs. non-random/random/usual placement	2	3	/	5	Gynell_2022; Wilson_2016	1	/	/	1	Meier_2022
Position: improved visibility of healthy products vs control stores	1	1	/	2	Harbers_2020; Shaw_2020	/	/	/	/	/
Position: proximity vs. increased distance control	5	/	/	5	Hollands_2019; Marcano-Olivier_2020; Laiou_2021; Wilson_2016	/	1	/	1	Bianchi_2018
Position: healthier/vegetarian default vs. less healthy/non-vegetarian default	3	/	/	3	Gynell_2022	3	/	/	3	Meier_2022
Presentation vs. usual/no action control	2	/	/	2	Laiou_2021; Metcalfe_2020	/	/	/	/	/
Size: smaller vs. larger	8	10	1	19	Al-Khudairy_2019; Chu_2021; Hollands_2015; Vargas-Alvarez_2021	1	/	/	1	Meier_2022
Information: Social norm message vs. no message/non-food message	1	1	/	2	Golding_2022	3*	6	1	10	Blackford_2021; Stiles_2022
Information: label vs. no label/active control	/	3	/	3	An_2021; Harbers_2020, Whatnall_2020	2**	/	/	2	Stiles_2022
Information: other (placemats, green arrows, public announcement) vs. control	2	/	/	2	Atanasova_2021; Golding_2022	/	1	/	1	Stiles_2022
Multicomponent vs. usual/no action control	3	/	/	3	Metcalfe_2020	/	/	/	/	/
Consumption (***n*** = 141)										
Availability higher vs. lower availability	1	3	/	4	Hollands_2019; Marcano-Olivier_2020	/	/	/	/	/
Position: healthier default vs. less healthy default	1	1	/	2	Gynell_2022	/	/	/	/	/
Position: proximity vs. increased distance control	8	8	/	16	Atanasova_2022; Hollands_2019; Marcano-Olivier_2020	/	/	/	/	/
Functionality vs. as usual control	2	1	/	3	Hollands 2015; Laiou_2021	/	/	/	/	/
Presentation vs. usual/no action control	2	1	/	3	Chu_2021; Hollands_2015; Laiou_2021	/	/	/	/	/
Size: smaller vs. larger	35	68	2	105	Hollands_2015; Vargas-Alvarez_2021	3	/	/	3	Bianchi_2018
Information: other (placemats) vs. no information control	1	/	/	1	Atanasova_2021	/	/	/	/	/
Information: label vs. no label	/	1	/	1	Hersey_2013	/	/	/	/	/
Multicomponent vs Usual/no action control	2	1	/	3	Funderburk_2020; Metcalfe_2020	/	/	/	/	/

*N* number of interventions;

* one of the interventions had an effect in women but not men.

** interventions had an effect in women but not men

#### Health

A meta-analysis (rated as “high” confidence) found a large effect size of lower availability of less healthy options reducing selection (*n* = 2/3 interventions included in our review) and a moderate reduction in consumption (*n* = 3/3), albeit with low certainty [66].

Only single studies from other reviews were included, showing mixed results: two suggesting positive effects (one hypothetical outcome [57], one selection/purchase [76]) and two suggesting no effect (one selection/purchase [77], and one selection and consumption [72]). All these reviews were rated either “low” [72, 77] or “critically low” [57, 76].

#### Sustainability

No studies were identified.

### Position

Ten reviews [57, 58, 64, 66, 67, 70, 72, 74, 78, 83] included 41 positioning interventions: 35 targeting health, 5 sustainability, and one both. Two reviews were rated “critically low” [57, 78], seven “low” [58, 64, 67, 70, 72, 74, 83], and one “high” confidence [66].

We sub-categorised positioning interventions into those manipulating Menus, Proximity, Defaults, and Visibility.

### Menus

#### Health

We included most menu placement studies from one review (*n* = 7/10 (70%)), which narratively summarised that the majority showed positive effects, although effect sizes varied from no to moderate effects [64]. One other review included a menu positioning study and found positive effects on purchases [83].

#### Sustainability

Two interventions from two reviews also found positive effects of menu placement interventions (on hypothetical outcomes [58] and purchases [74]).

### Proximity

#### Health

A meta-analysis found that decreased proximity to less healthy options reduces consumption (*n* = 12/15) [66]. Four other studies also supported this (two measuring consumption and two selection [66]).

Single studies from other reviews similarly found positive effects (on selection (*n* = 3) [70, 72, 83] and consumption (*n* = 1) [57]), with the exception of one study, which found no effect [72] on consumption.

#### Sustainability

Only one study was identified, which did not find an effect [58].

### Defaults

#### Health

We included three of seven default studies from one review, which concluded that whilst defaults appear to encourage healthier food selections, the effects on consumption are less clear [64].

#### Sustainability

We included most default studies from another review (7/9) [74], where the authors concluded that defaults reduce meat consumption [74] (N.B. three of these were also identified in another review but not categorised as default interventions there [58], and one of which we classified as *menu placement* rather than default).

### Increased visibility

#### Health

Two studies from two reviews found no [78] or positive effects of increased visibility on purchases [67].

#### Sustainability

No studies were identified.

### Presentation

Eight reviews included 17 presentation interventions [58, 60, 64, 65, 69, 70, 73, 75], 10 targeting health, and 7 sustainability. Four reviews were rated “critically low” [60, 69, 73, 75], three “low” [58, 64, 70], and one “high” [65]. Most (6/10) health interventions targeted children, whilst all sustainability interventions targeted adults.

#### Health

Studies reported mostly positive results. Two studies from one review found redesigned menus encouraged healthier hypothetical food selections [64], whilst studies manipulating food shape found positive effects on purchase (*n* = 1) [75] and consumption (*n* = 2) [65, 70]. Interventions changing packaging found positive effects on selection or purchase requests (*n* = 3) [69, 70] but no effect on consumption (*n* = 1) [60] or hypothetical outcomes (*n* = 1) [69].

#### Sustainability

Results were mixed, with three of four interventions from one review finding positive effects of changing meal presentation [58], but in another review, three interventions found no effect [73].

### Functionality

Two reviews [65, 70] included three functionality interventions, with reviews rated “high” [65] and “low” [70] confidence.

#### Health

Two interventions from one review resulted in positive consumption effects [70], whilst another found no effect [65].

#### Sustainability

No studies were identified.

### Size

Six reviews investigated 118 size interventions [55, 58, 60, 65, 74, 81], of which 114 targeted health, two sustainability, and two both. Most interventions (*n* = 105) were from a high-quality Cochrane review [65], whilst the remaining reviews were rated “moderate” [55], “low” [58, 74], or “critically low” [60, 81] confidence.

A meta-analysis of size interventions found that smaller sizes decrease selection (*n* = 11/13) and consumption (*n* = 84/92), with small to moderate relative effect sizes for both [65].

We differentiate interventions into Tableware, Portions, Packages, Individual units, or a Combination of size interventions.

### Tableware

#### Health

Two meta-analyses with considerable overlap found that smaller tableware reduced consumption and selection (Cochrane review: (*n* = 12/12) and (*n* = 7/7) respectively [65]; Systematic review: consumption (*n* = 18/21) and selection (*n* = 6/7) [81]). One other intervention identified in the Cochrane review found that smaller sizes reduced selection [65], whilst two other studies found no or a negative effect on selection [81].

### Sustainability

No studies were identified.

### Portions

#### Health

A meta-analysis found smaller portion sizes reduce consumption (*n* = 58/58) and selection (*n* = 3/5) [65]. Eight other interventions identified in the Cochrane review mostly found smaller portions reduced consumption (*n* = 5), with two finding no effects and one a negative effect [65]. Two studies from other reviews found positive effects on purchases [55] or consumption [81].

#### Sustainability

One review found that reducing meat portions lowers meat consumption [58], although two of three interventions overlapped with another review [65]. Stocking a smaller portion size of sausages in a store reduced meat sales [74].

### Packages

#### Health

A meta-analysis found that smaller packages reduced consumption (*n* = 9/10, although we characterised one intervention as functionality rather than size) [65]. Two other interventions from the Cochrane review recorded no effect on consumption or a positive effect on selection [65], whilst in contrast another intervention from a different review found no effect on selection [60].

#### Sustainability

No studies were identified.

### Individual units

#### Health

One review [67] found no effect on food selection (*n*=3). Similarly, two other interventions found that smaller individual unit size reduced consumption whilst the majority (*n*=5) found no effect.

#### Sustainability

No studies were identified.

### Combination of size interventions

#### Health

Three interventions from one review [65] manipulated package and individual unit size and found this reduced consumption.

#### Sustainability

No studies were identified.

### Information

Fifteen reviews included 35 information interventions [13, 51, 56–59, 62–64, 67, 68, 71, 79, 80, 82], 18 targeting health and 17 targeting sustainability. Most were rated “low” confidence [58, 59, 62, 64, 67, 68, 79, 82] (*n* = 8), followed by “critically low” (*n* = 6) [13, 51, 56, 57, 63, 71] and “moderate” (*n* = 1) [80].

We included four of five labelling or description interventions from a sustainability-focused review, which found these overall did not lower meat demand [58]. We sub-categorise information interventions into Labels, Social norms, or Changed food dish descriptions.

### Labels

#### Health

*Calling out healthy options*: A green tick label improved a hypothetical outcome in one study [68], but in another, a healthy choice tick did not [51]. Similarly, in three other studies, healthy choices logos did not affect hypothetical outcomes or purchases [51, 56, 67].

*Labelling all items*: A health star label positively affected a hypothetical outcome [63] but neither this nor a healthiness star rating labels were shown to impact on purchases [56, 82]. Two studies investigating 5-colour nutrition labels found positive and no effects on hypothetical outcomes [56, 68], whilst two nutriscore label interventions found no difference [71, 79]. In contrast, a NuVal label resulted in significant improvements of a hypothetical outcome [59].

#### Sustainability

Two labels calling out the more sustainable options increased sustainable food purchases for women but not men [80]. A leaf symbol did not have an effect on a hypothetical outcome [58].

### Social norms

#### Health

One review included two social norms interventions, with one finding positively influenced purchases and one no effect [62].

#### Sustainability

These results were also mixed: A review found that two of five dynamic norm interventions increased vegetarian sales, with two finding no effect and one intervention discouraging vegetarian orders [80]. One static norm intervention showed no difference to control, whilst a label implying a social norm (“Student choice”) increased vegetarian purchases in women but not men [80]. Three injunctive social norm interventions from another review found no effect on purchases in a factorial RCT [13].

### Changed food dish descriptions 

#### Health

One study from one review found that changing names of healthy dishes to use more descriptive language did not affect a hypothetical outcome [64].

#### Sustainability

Three interventions from one review did not affect a hypothetical outcome [58].

### Other

#### Health

Using green arrows to signpost store customers towards healthier choices increased healthy purchases [62]. Placemats promoting healthy kids meals in a restaurant were also found to increase purchases and intake of healthier meals [57].

#### Sustainability

Advertising bean dishes via school announcement systems had no effect on overall selection of these dishes [80].

### Multicomponent

Four interventions from two reviews [61, 75] investigated multi-component interventions. Both reviews were rated “critically low” confidence [56, 61, 75].

#### Health

Two interventions combining positioning, presentation, availability and information or positioning, presentation and information found positive effects on selection, with the former also finding increased consumption, whilst the latter found no difference [75]. Two interventions combined availability, positioning and information, finding reduced energy purchases or intake [61, 75].

#### Sustainability

No studies were identified.

### Mediators, moderators and cross-cultural factors

Limited evidence was identified for mediators, moderators and cross-cultural factors relating to micro-environmental interventions, with no comparable evidence for health vs. sustainability. The identified factors did not suggest particular potential for these to differentially impact healthy and sustainable food targets. These factors are narratively summarised in Appendix 5, subdivided into those relating to intervention characteristics and those relating to participant characteristics. Although five reviews [65–68, 74] included investigating moderators and mediators as a review aim, most reviews were primarily focused on evaluating effectiveness and did not aim to specifically investigate mediators or moderators.

### Risk of bias

Overall, the quality of reviews, assessed using AMSTAR2 [53], was poor (Table 5). Solely two Cochrane reviews received a “high” confidence rating [65, 66] and two other reviews were rated as “moderate” [55, 80]. Of the remaining reviews, 13 received a “low” [58, 59, 62, 64, 67, 68, 70, 72, 74, 77, 79, 82, 83] and 14 a “critically low” rating [13, 51, 56, 57, 60, 61, 63, 69, 71, 73, 75, 76, 78, 81]. Regarding critical domains, reviews most commonly did not account for risk of bias when discussing results (*n* = 21) [13, 51, 56, 58, 60–64, 67–71, 74, 76–78, 81–83] or did not mention a pre-defined protocol (*n* = 13) [13, 51, 56, 57, 59–61, 63, 69, 71, 72, 75, 76]. The most commonly unmet non-critical domains were not providing funding details of included studies (*n* = 29) [13, 51, 55–64, 67–83], not providing a list of excluded studies with reason (*n* = 27) [13, 51, 55–64, 67–73, 75–78, 80–83], and insufficient discussion of heterogeneity (*n* = 22) [13, 51, 55, 58, 60–64, 67–72, 75–78, 80, 82, 83]. In contrast, all but two reviews had at least a partial yes for the comprehensiveness of the literature search [51, 81] and providing detailed descriptions of included studies [13, 77].

**Table 5:** AMSTAR2 [53] risk of bias assessment

Review ID	1	2*	3	4*	5	6	7	8	9*	10	11*	12	13*	14	15*	16	Confidence rating
Al-Khudairy_2019 [55]	N	PY	N	PY	Y	Y	N	PY	Y, PY	N	N/A	N/A	Y	N	N/A	Y	Moderate
An_2021 [56]	N	N	N	PY	Y	N	N	PY	N, N	N	N/A	N/A	N	Y	N/A	Y	Critically low
Atanasova_2022 [57]	Y	N	Y	PY	Y	N	N	PY	N, PY	N	N/A	N/A	Y	Y	N/A	N	Critically low
Bianchi_2018 [58]	Y	PY	Y	PY	Y	Y	N	PY	PY, PY	N	N/A	N/A	N	N	N/A	Y	Low
Blackford_2021 [13]	N	N	N	PY	N	N	N	N	N, N	N	N/A	N/A	N	N	N/A	N	Critically low
Cameron_2016 [59]	N	N	N	PY	N	N	N	Y	PY, PY	N	N/A	N/A	Y	Y	N/A	N	Low
Chu_2021 [60]	N	N	N	PY	Y	N	N	PY	PY, PY	N	N/A	N/A	N	N	N/A	Y	Critically low
Funderburk_2020 [61]	N	N	N	PY	N	N	N	PY	N, N	N	N/A	N/A	N	N	N/A	Y	Critically low
Golding_2022 [62]	Y	Y	N	PY	N	Y	N	Y	PY, PY	N	N/A	N/A	N	N	N/A	Y	Low
Gupta_2021 [63]	Y	N	N	PY	N	Y	N	Y	PY, PY	N	N/A	N/A	N	N	N/A	Y	Critically low
Gynell_2022 [64]	Y	PY	N	PY	Y	N	N	PY	PY, PY	N	N/A	N/A	N	N	N/A	Y	Low
Harbers_2020 [67]	N	PY	N	PY	Y	N	N	PY	PY, PY	N	N/A	N/A	N	N	N/A	Y	Low
Hartmann-Boyce_2018 [68]	Y	Y	Y	PY	Y	Y	N	PY	Y	N	N/A	N/A	N	N	N/A	Y	Low
Hersey_2013 [51]	N	N	N	N	N	N	N	PY	N, N	N	N/A	N/A	N	N	N/A	Y	Critically low
Hollands_2015 [65]	Y	Y	Y	PY	Y	Y	Y	Y	Y	Y	Y	Y	Y	Y	Y	N	High
Hollands_2019 [66]	Y	PY	Y	PY	Y	Y	Y	Y	Y	Y	Y	N	Y	Y	Y	Y	High
Kraak_2015 [69]	N	N	N	PY	Y	Y	N	PY	N, N	N	N/A	N/A	N	N	N/A	Y	Critically low
Laiou_2021 [70]	Y	PY	Y	PY	N	Y	N	Y	Y, PY	N	N/A	N/A	N	N	N/A	N	Low
Ljusic_2022 [71]	N	N	N	PY	Y	N	N	PY	Y, PY	N	N/A	N/A	N	N	N/A	Y	Critically low
Marcano-Olivier_2020 [72]	Y	N	N	PY	N	N	N	Y	PY, PY	N	N/A	N/A	Y	N	N/A	N	Low
Mathur_2021 [73]	Y	PY	N	PY	Y	N	N	PY	N, N	N	N	N	Y	Y	Y	Y	Critically low
Meier_2022 [74]	Y	PY	N	PY	N	Y	Y	Y	PY, Y	N	N/A	N/A	N	Y	N/A	Y	Low
Metcalfe_2020 [75]	Y	N	N	PY	N	Y	N	PY	N, PY	N	N/A	N/A	Y	N	N/A	Y	Critically low
Osei-Assibey_2012 [76]	N	N	N	PY	N	Y	N	PY	PY, PY	N	N/A	N/A	N	N	N/A	Y	Critically low
Richardson_2022 [77]	N	PY	N	PY	Y	N	N	N	Y, Y	N	N/A	N/A	N	N	N/A	Y	Low
Shaw_2020 [78]	N	PY	Y	PY	N	Y	N	PY	N, N	N	N/A	N/A	N	N	N/A	Y	Critically low
Slapø_2021 [79]	Y	Y	Y	PY	Y	N	Y	Y	Y	N	N	N	Y	Y	Y	Y	Low
Stiles_2022 [80]	N	PY	N	PY	Y	N	N	PY	PY, PY	N	N/A	N/A	Y	N	N/A	Y	Moderate
Vargas-Alvarez_2021 [81]	Y	PY	N	N	Y	Y	N	Y	N, N	N	N	N	N	Y	Y	Y	Critically low
Whatnall_2020 [82]	Y	Y	N	PY	Y	Y	N	Y	PY, PY	N	N/A	N/A	N	N	N/A	Y	Low
Wilson_2016 [83]	Y	Y	Y	PY	Y	N	N	PY	PY, PY	N	N/A	N/A	N	N	N/A	N	Low

* critical domain. AMSTAR 2 items [53]: (1) Fulfilment of PICO criteria; (2) Written protocol and any deviations from protocol; (3) Explanation of selection of study designs; (4) Comprehensiveness of search strategy; (5) Duplicate study selection; (6) Duplicate data extraction; (7) Listed excluded studies and exclusion reason; (8) Detailed description of included studies; (9) Satisfactory RoB assessment for RCTs, NRSI; (10) Funding sources of included studies; (11) Appropriateness of meta-analysis; (12) RoB impact assessment in meta-analyses; (13) RoB considered in interpretation/discussion of results; (14) Heterogeneity explained/discussed; (15) Publication bias assessment for meta-analyses; (16) Conflict of interest (yes means no conflict of interest or management of any conflict of interest). Y = yes; PY = partial yes; N = no. CL = Critically Low. “Yes” means the answer is favourable, whilst “no” denotes a negative result or the absence of information. Reviews receive a “high” confidence rating if they do not have more than one non-critical weakness, “moderate” if they exceed one non-critical weakness, “low” if there is one critical flaw, and “critically low” if they exceed one critical flaw

## Discussion

We found that there is less evidence for micro-environmental interventions aiming to encourage more sustainable dietary behaviours (*n* = 31) compared to healthier behaviours (*n* = 194). Whilst interventions aimed at healthier dietary behaviours were mostly size (*n* = 116 (60%)) and positioning (*n* = 36 (19%)) interventions, the majority of sustainable food interventions were information (*n* = 17 (55%)) and presentation (*n* = 7 (23%)) interventions. Overlap between health and sustainability interventions was limited: of 27 subcomponents (Table 4), only 5 (defaults, size, presentation, labels, and social norms) have multiple identified interventions for both health and sustainability. Given these low numbers of overlapping intervention targets, it is difficult to draw conclusions as to the comparability of effects for health versus sustainability.

We found the most promising evidence for position and size interventions, which appear effective for improving both health and sustainability. However, findings for health interventions are more robust than for sustainability due to a much larger number of health-focused interventions and two included Cochrane reviews specifically focusing on these intervention types. Defaults, menu positioning, and reduced portion sizes showed positive results for health and sustainability, although this is based on a small number of sustainability interventions. Proximity interventions were successful for healthier foods, but only investigated by one sustainability intervention which found no effect. We did not find any sustainability interventions testing other operationalisations of size interventions (Tableware, Packages, Combination of size interventions), but these appear successful for health-focused interventions.

Furthermore, we identified potentially promising evidence of presentation interventions for health and sustainability. However, most health studies targeted children, whilst all sustainability studies involved adults, limiting comparability. Moreover, for health, 50% of presentation interventions were hypothetical, and for sustainability-focused interventions, only hypothetical outcomes were measured, limiting the conclusions that can be drawn. This may reflect that reducing consumption of a food (often meat for sustainability, or less healthy options) by adding images with negative connotations may be less feasible or acceptable to test in real-world settings, resulting in researchers often using hypothetical outcomes.

The evidence base for information interventions was comparable in size but not proportion for health (*n* = 18 (9%)) and sustainability interventions (*n* = 17 (55%)). The potential for social norms and label interventions to encourage healthier or more sustainable food behaviour appears limited. Additionally, changing the description of food dishes did not impact measured outcomes for both health and sustainability. Despite social norms being the most commonly investigated type of information intervention for sustainability, most social norms studies (5/7) were conducted by the same first author. The popularity of information interventions for sustainability studies may be linked to the higher acceptability of information interventions, such as labels, compared to more intrusive interventions, such as decreasing availability [84]. However, the evidence identified here suggests limited effectiveness of information interventions to encourage more sustainable food choices.

Sizeable gaps remain in the evidence base. Availability, functionality, and multi-component interventions appear promising based on a small number of health-focused studies, but were not investigated for sustainability and therefore no comparisons could be made. Additionally, a higher proportion of interventions with hypothetical outcomes were identified overall for sustainability (12/31 (39%)) compared to 24/194 (12%) for health, demonstrating a lack of real-world trials of interventions aiming to encourage more sustainable choices. This may reflect the relative newness of this field or a higher level of difficulty to conduct real-world studies. Sustainability-focused studies often targeted meat, potentially making interventions less acceptable [84] and more easily apparent to consumers. Existing evidence shows low acceptance of meat-reduction interventions among caterers due to customer satisfaction concerns [85] and lower meal participation and higher plate waste on vegetarian days in Finnish schools shortly after introduction [86]. This also could mean that currently sustainability interventions are focused primarily on reduction, whilst healthier interventions may be more split between increasing healthier and reducing less healthy food options. Increasing vs decreasing options can have different behavioural effects [87], with interventions targeting reducing selection or consumption tending to be more restrictive [88]. Further research aimed at increasing more sustainable food consumption could fill this research gap.

There was a scarcity of data on mediators, moderators, and cross-cultural factors across reviews. This could reflect an actual lack of evidence—particularly given the low numbers of identified studies for sustainability. This would be in line with previous evidence [39], including reviews specifically aiming to assess effect modifiers—in particular socioeconomic position—being limited by the scarcity of such information [65–68]. Alternatively, this might be due to lack of reporting within the systematic reviews, given most included systematic reviews primarily aimed to investigate the effectiveness of interventions. Although we did not assess this systematically, we are aware of at least one primary study exploring cross-cultural factors [89] where this was not directly reported in the review [58]. In terms of cross-cultural factors, relatively homogeneous study populations within reviews (e.g. all bar two reviews were conducted exclusively by researchers from high-income countries and the vast majority of individual studies were from high-income countries) may mean such factors were not explored. The relative neglect of both mechanism and cross-cultural factors in the evidence base for interventions to change dietary behaviour limits our ability to hypothesise and establish contextual factors that may determine relative effectiveness.

Whilst the current evidence bases make it difficult to determine the extent to which health and sustainability interventions could be expected to result in similar effects, in practice, it is clear that health and sustainability goals can overlap. For example, interventions aimed at healthier (i.e. smaller) portion sizes could have environmental co-benefits if they lower food consumption without increasing food waste, and sustainability-motivated interventions focused on increasing more sustainable choices could have health benefits (and vice versa), given links between the sustainability and healthiness of foods [2, 3]. However, further evidence is needed before we can determine if these evidence bases can be fully integrated. For example, the framing of these interventions might impact public support and subsequent effectiveness [90].

This overview of reviews focused on micro-environmental interventions, which may present a relatively acceptable strategy to the public as opposed to more restrictive measures such as advertising bans or price increases [84]. Nevertheless, whilst micro-environmental interventions can play a part in encouraging dietary change—particularly when implemented at scale—widespread systemic changes are required to keep the food system in line with planetary boundaries [91], encompassing dietary changes, technological improvements, and lowered food waste [92]. Moreover, even if not directly implemented as a policy, upstream changes, such as the mandatory reporting and health targets announced by the government in England [93], may lead to industry looking to make effective changes to microenvironments (e.g. availability or portion size).

Strengths of this review include that we only included RCTs, as they minimise the risk of confounders [94], and focused purely on micro-environmental interventions following, with minor adaptations, the established TIPPME taxonomy [11], enabling us to isolate the effect of micro-environmental interventions. We give a broad overview of the effectiveness of six different types of micro-environmental interventions, with more detailed subcategories for the more well-researched categories, illustrating the current evidence base and gaps. We separated out impacts of hypothetical vs. real-world outcomes, given the potential for different behavioural responses.

Our overview of reviews has several limitations. As we primarily extracted at review level, we are relying on the accurate presentation of study characteristics and results within identified reviews. This may have affected study categorisation into intervention type, and we acknowledge that others may have categorised some of these differently. This also means that we were not always able to obtain effect sizes, so that whilst we can identify the consistency with which, e.g. positive effects were found, we were unable to consider the relative effect sizes of the interventions in this review. Furthermore, we did not differentiate between subjective and objective outcome measurements. Whilst self-reported outcomes can be prone to reporting bias [95], this is unlikely to have substantially impacted our findings, given the majority of the included studies from reviews relied on objective outcome measurements [e.g. 65, 66]. As our review is an overview of reviews, publication bias could have affected our findings both at the review level (i.e. bias in the reviews being published) and at the study level (i.e. bias in the studies being published, and therefore available to be included in a review). Given our aim to compare across health and sustainability dietary outcomes, it would be of particular concern if publication bias differentially impacted on these outcomes for similar interventions. There was little evidence of publication bias from (relatively few) formal assessments in previous reviews, and we believe that the risk of publication bias significantly affecting our results is mitigated by our comprehensive search and large number of included reviews and studies. Nevertheless, assessment of the relative levels of publication bias for health vs. sustainability interventions would be beneficial in future research. We did not conduct additional grey literature searches. However, some of the searched databases included grey literature and it is unlikely that grey literature reports would have met our inclusion criteria (e.g. definition for systematic reviews). Duplicate screening and extraction for the forward and backward citation searches was only conducted for a subset of studies [96], though agreement for this subset was good. Whilst we only included systematic reviews where results from included RCTs could be isolated, we are aware that particularly in real-world food environments, conducting RCTs is not always feasible and we may have missed good-quality non-randomised studies in real-world food environments.

Evidence for interventions aiming to encourage more sustainable diets is scarce, and there is little overlap of health and sustainability interventions in terms of intervention type. Therefore, we are not in a position to draw conclusions regarding the extent to which health interventions can directly inform the likely effectiveness of sustainability interventions, or vice versa. More comparable evidence is needed, prioritising those interventions that seem most promising for health (e.g. size, position), as there currently is no evidence that health and sustainability interventions respond differently. Additionally, more evidence is required for intervention categories that have received less research attention but appear promising, such as functionality, presentation, and availability interventions, and multicomponent combinations of intervention types.

The vast majority of evidence included comes from high-income countries. Given the lack of data from lower-income countries, evidence may not be transferable to these settings, although high-income countries in particular need to drastically reduce their carbon footprint [97]. Nevertheless, more evidence from low- and middle-income countries is needed to ascertain the health equity impacts of micro-environmental interventions within and across different socio-economic and cultural contexts.

In particular, more real-world studies with non-hypothetical outcomes are needed, especially for sustainability. Although setting up and conducting field trials can be challenging [98], our review included numerous field trials showing such trials are feasible, and guidance on conducting field trials is available in the literature [99–101]. Additionally, meta-analyses comparing health and environmental sustainability, particularly if broken down by intervention type due to the heterogeneous nature of micro-environmental interventions, could give important directions for future research. Future research should also investigate moderators and mediators of micro-environmental interventions aiming to encourage healthier or more sustainable dietary behaviour. Investigating both effectiveness and potential mediators and moderators of micro-environmental interventions will generate a more comprehensive assessment of intended and unintended effects of interventions as well as providing opportunity to optimise intervention strategies.

## Conclusions

Healthier and more environmentally sustainable diets are needed for our planetary health, with micro-environmental changes potentially providing one piece of this puzzle. This review set out to compare evidence for health vs. environmental sustainability interventions to assess whether these might be integrated into a unified evidence base and enhance our predictive power at selecting the most promising interventions. However, the review highlights the limited evidence base for micro-environmental interventions encouraging more environmentally sustainable dietary behaviour. Limited overlap between interventions for health and environmental sustainability hindered robust judgements of the extent to which the evidence for health-focused dietary interventions transfers to environmental sustainability-focused dietary interventions. At present, evidence for default, size and menu positioning interventions is promising and consistent for both health and environmental sustainability, albeit based off limited studies. One option to potentially accelerate change would be to explore using what the current review has identified as a larger and more mature evidence base for health interventions to inform which sustainability interventions should be prioritised—i.e. those interventions that are most promising for health.

## Supplementary Information

Supplementary Material 1. Search strategy.

Supplementary Material 2. Review selection & data extraction.

Supplementary Material 3. Examples of information interventions.

Supplementary Material 4. Detailed overview of identified studies.

Supplementary Material 5. Mediators, moderators and cross-cultural factors.

## Data Availability

No datasets were generated or analysed during the current study.

## Abbreviations

AMSTAR2: A MeaSurement Tool to Assess systematic Reviews
PICOS: Population, Intervention, Comparison, Outcomes, and Study design
PRIOR: Preferred reporting items for overviews of reviews
RCT: Randomised controlled trial
TIPPME: Typology of interventions in proximal physical micro-environments

## References

[1] Springmann M, Wiebe K, Mason-D’Croz D, Sulser TB, Rayner M, Scarborough P. Health and nutritional aspects of sustainable diet strategies and their association with environmental impacts: a global modelling analysis with country-level detail. Lancet Planet Health. 2018;2(10):e451-61. 30318102 10.1016/S2542-5196(18)30206-7 PMC6182055

[2] Clark M, Springmann M, Rayner M, Scarborough P, Hill J, Tilman D, et al. Estimating the environmental impacts of 57,000 food products. Proc Natl Acad Sci U S A. 2022;119(33):e2120584119. 35939701 10.1073/pnas.2120584119 PMC9388151

[3] Clark MA, Springmann M, Hill J, Tilman D. Multiple health and environmental impacts of foods. Proc Natl Acad Sci. 2019;116(46):23357–62. 31659030 10.1073/pnas.1906908116 PMC6859310

[4] Scarborough P, Clark M, Cobiac L, Papier K, Knuppel A, Lynch J, et al. Vegans, vegetarians, fish-eaters and meat-eaters in the UK show discrepant environmental impacts. Nat Food. 2023;4(7):565–74. 37474804 10.1038/s43016-023-00795-w PMC10365988

[5] Martínez-González MA, Salas-Salvadó J, Estruch R, Corella D, Fitó M, Ros E. Benefits of the Mediterranean diet: insights from the PREDIMED study. Prog Cardiovasc Dis. 2015;58(1):50–60. 25940230 10.1016/j.pcad.2015.04.003

[6] Poore J, Nemecek T. Reducing food’s environmental impacts through producers and consumers. Science. 2018;360(6392):987–92. 29853680 10.1126/science.aaq0216

[7] Boada LD, Henríquez-Hernández LA, Luzardo O. The impact of red and processed meat consumption on cancer and other health outcomes: epidemiological evidences. Food Chem Toxicol. 2016;92:236–44. 27106137 10.1016/j.fct.2016.04.008

[8] Wang X, Lin X, Ouyang YY, Liu J, Zhao G, Pan A, et al. Red and processed meat consumption and mortality: dose–response meta-analysis of prospective cohort studies. Public Health Nutr. 2016;19(5):893–905. 26143683 10.1017/S1368980015002062 PMC10270853

[9] Ritchie H, Roser M. Meat and Dairy Production: Our World in Data 2017 [updated 2019]. Available from: https://ourworldindata.org/meat-production.

[10] Graça J, Godinho CA, Truninger M. Reducing meat consumption and following plant-based diets: current evidence and future directions to inform integrated transitions. Trends Food Sci Technol. 2019;91:380–90.

[11] Hollands GJ, Bignardi G, Johnston M, Kelly MP, Ogilvie D, Petticrew M, et al. The TIPPME intervention typology for changing environments to change behaviour. Nat Hum Behav. 2017;1:0140.

[12] Abrahamse W. How to effectively encourage sustainable food choices: a mini-review of available evidence. Front Psychol. 2020;11:3134. 10.3389/fpsyg.2020.589674 PMC7701282 33304299

[13] Blackford B. Nudging interventions on sustainable food consumption: a systematic review. The Journal of Population and Sustainability. 2021;5(2):17–62–17–62.

[14] Ronto R, Saberi G, Leila Robbers GM, Godrich S, Lawrence M, Somerset S, et al. Identifying effective interventions to promote consumption of protein-rich foods from lower ecological footprint sources: a systematic literature review. PLoS Glob Public Health. 2022;2(3):e0000209. 36962370 10.1371/journal.pgph.0000209 PMC10021177

[15] Vecchio R, Cavallo C. Increasing healthy food choices through nudges: a systematic review. Food Qual Prefer. 2019;78:103714.

[16] Mertens S, Herberz M, Hahnel UJ, Brosch T. The effectiveness of nudging: a meta-analysis of choice architecture interventions across behavioral domains. Proc Natl Acad Sci U S A. 2022;119(1):e2107346118. 34983836 10.1073/pnas.2107346118 PMC8740589

[17] Crippa M, Solazzo E, Guizzardi D, Monforti-Ferrario F, Tubiello FN, Leip A. Food systems are responsible for a third of global anthropogenic GHG emissions. Nat Food. 2021;2:198–209. 37117443 10.1038/s43016-021-00225-9

[18] Intergovernmental Panel on Climate Change (IPCC). Summary for Policymakers. 2021. In: Climate Change 2021: The Physical Science Basis Contribution of Working Group I to the Sixth Assessment Report of the Intergovernmental Panel on Climate Change. Cambridge University Press; [3–32]. Available from: https://www.ipcc.ch/report/ar6/wg2/chapter/summary-for-policymakers/.

[19] Mullee A, Vermeire L, Vanaelst B, Mullie P, Deriemaeker P, Leenaert T, et al. Vegetarianism and meat consumption: a comparison of attitudes and beliefs between vegetarian, semi-vegetarian, and omnivorous subjects in Belgium. Appetite. 2017;114:299–305. 28392424 10.1016/j.appet.2017.03.052

[20] Pechey R, Hollands GJ, Marteau TM. Explaining the effect on food selection of altering availability: two experimental studies on the role of relative preferences. BMC Public Health. 2022;22(1):868. 35501746 10.1186/s12889-022-13067-2 PMC9063226

[21] Piazza J, Ruby MB, Loughnan S, Luong M, Kulik J, Watkins HM, et al. Rationalizing meat consumption. The 4Ns. Appetite. 2015;91:114–28. 25865663 10.1016/j.appet.2015.04.011

[22] Hartmann C, Siegrist M. Consumer perception and behaviour regarding sustainable protein consumption: a systematic review. Trends Food Sci Technol. 2017;61:11–25.

[23] Sanchez-Sabate R, Sabaté J. Consumer attitudes towards environmental concerns of meat consumption: a systematic review. Int J Environ Res Public Health. 2019;16(7):1220. 30959755 10.3390/ijerph16071220 PMC6479556

[24] Lappalainen R, Kearney J, Gibney M. A pan EU survey of consumer attitudes to food, nutrition and health: an overview. Food Qual Prefer. 1998;9(6):467–78.

[25] Paquette M-C. Perceptions of healthy eating: state of knowledge and research gaps. Canadian Journal of Public Health= Revue canadienne de sante publique. 2005;96(Suppl 3):S16.16042159

[26] Blanke J, Billieux J, Vögele C. Healthy and Sustainable Food Shopping: A Survey of Intentions and Motivations. Front Nutr. 2022;9:742614. 10.3389/fnut.2022.742614 PMC8924458 35308289

[27] Baudry J, Péneau S, Allès B, Touvier M, Hercberg S, Galan P, et al. Food choice motives when purchasing in organic and conventional consumer clusters: focus on sustainable concerns (the NutriNet-Santé cohort study). Nutrients. 2017;9(2):88. 28125035 10.3390/nu9020088 PMC5331519

[28] Hoek AC, Pearson D, James SW, Lawrence MA, Friel S. Shrinking the food-print: a qualitative study into consumer perceptions, experiences and attitudes towards healthy and environmentally friendly food behaviours. Appetite. 2017;108:117–31. 27686818 10.1016/j.appet.2016.09.030

[29] Worsley A. Nutrition knowledge and food consumption: can nutrition knowledge change food behaviour? Asia Pac J Clin Nutr. 2002;11:S579–85. 12492651 10.1046/j.1440-6047.11.supp3.7.x

[30] Wardle J, Haase AM, Steptoe A, Nillapun M, Jonwutiwes K, Bellisie F. Gender differences in food choice: the contribution of health beliefs and dieting. Ann Behav Med. 2004;27:107–16. 15053018 10.1207/s15324796abm2702_5

[31] Grundy EAC, Slattery P, Saeri AK, Watkins K, Houlden T, Farr N, et al. Interventions that influence animal-product consumption: a meta-review. Future Foods. 2022;5:100111.

[32] Wolfenden L, Barnes C, Lane C, McCrabb S, Brown HM, Gerritsen S, et al. Consolidating evidence on the effectiveness of interventions promoting fruit and vegetable consumption: an umbrella review. Int J Behav Nutr Phys Act. 2021;18(1):11. 33430879 10.1186/s12966-020-01046-y PMC7798190

[33] Gupta A, Alston L, Needham C, Robinson E, Marshall J, Boelsen-Robinson T, et al. Factors influencing implementation, sustainability and scalability of healthy food retail interventions: a systematic review of reviews. Nutrients. 2022. 10.3390/nu14020294. 35057476 10.3390/nu14020294 PMC8780221

[34] Løvhaug AL, Granheim SI, Djojosoeparto SK, Harrington JM, Kamphuis CBM, Poelman MP, et al. The potential of food environment policies to reduce socioeconomic inequalities in diets and to improve healthy diets among lower socioeconomic groups: an umbrella review. BMC Public Health. 2022;22(1):433. 35246074 10.1186/s12889-022-12827-4 PMC8895543

[35] Brand T, Pischke CR, Steenbock B, Schoenbach J, Poettgen S, Samkange-Zeeb F, et al. What works in community-based interventions promoting physical activity and healthy eating? A review of reviews. Int J Environ Res Public Health. 2014;11(6):5866–88. 24886756 10.3390/ijerph110605866 PMC4078553

[36] Capper TE, Brennan SF, Woodside JV, McKinley MC. What makes interventions aimed at improving dietary behaviours successful in the secondary school environment? A systematic review of systematic reviews. Public Health Nutr. 2022;25(9):2448–64. 35357283 10.1017/S1368980022000829 PMC9991643

[37] Matwiejczyk L, Mehta K, Scott J, Tonkin E, Coveney J. Characteristics of effective interventions promoting healthy eating for pre-schoolers in childcare settings: an umbrella review. Nutrients. 2018. 10.3390/nu10030293. 29494537 10.3390/nu10030293 PMC5872711

[38] Verdonschot A, Follong BM, Collins CE, de Vet E, Haveman-Nies A, Bucher T. Effectiveness of school-based nutrition intervention components on fruit and vegetable intake and nutrition knowledge in children aged 4–12 years old: an umbrella review. Nutr Rev. 2023;81(3):304–21. 35947869 10.1093/nutrit/nuac057 PMC9912007

[39] Szaszi B, Palinkas A, Palfi B, Szollosi A, Aczel B. A systematic scoping review of the choice architecture movement: toward understanding when and why nudges work. J Behav Decis Making. 2018;31(3):355–66.

[40] Gates M, Gates A, Pieper D, Fernandes RM, Tricco AC, Moher D, et al. Reporting guideline for overviews of reviews of healthcare interventions: development of the PRIOR statement. BMJ. 2022. 10.1136/bmj-2022-070849. 35944924 10.1136/bmj-2022-070849 PMC9361065

[41] Centre for Reviews and Dissemination. CRD's guidance for undertaking reviews in healthcare: York Publ. Services; 2009. Available from: https://www.york.ac.uk/media/crd/Systematic_Reviews.pdf.

[42] FSS. Briefing paper on discretionary foods. Food Standards Scotland; 2018. Available online from: https://www.foodstandards.gov.scot/downloads/FSS_-_Discretionary_Foods_Paper_-_September_2018_final_for_publication.pdf.

[43] Haddaway NR, Grainger MJ, Gray CT. Citationchaser: a tool for transparent and efficient forward and backward citation chasing in systematic searching. Res Synth Methods. 2022;13(4):533–45. 35472127 10.1002/jrsm.1563

[44] Covidence. Covidence systematic review software Melbourne, Australia: Veritas Health Innovation; 2022. Available from: www.covidence.org.

[45] Pollock M, Fernandes RM, Newton AS, Scott SD, Hartling L. A decision tool to help researchers make decisions about including systematic reviews in overviews of reviews of healthcare interventions. Syst Rev. 2019;8(1):29. 30670086 10.1186/s13643-018-0768-8 PMC6341524

[46] Pollock M, Fernandes RM, Becker LA, Pieper D, Hartling L. Chapter V: Overviews of Reviews. 2022. In: Cochrane Handbook for Systematic Reviews of Interventions. Cochrane. 6.3. Available from: https://training.cochrane.org/handbook/current/chapter-v.

[47] Aromataris E, Fernandez R, Godfrey CM, Holly C, Khalil H, Tungpunkom P. Summarizing systematic reviews: methodological development, conduct and reporting of an umbrella review approach. JBI Evid Implement. 2015;13(3):132–40. 10.1097/XEB.0000000000000055 26360830

[48] Hollands GJ, Marteau TM, Fletcher PC. Non-conscious processes in changing health-related behaviour: a conceptual analysis and framework. Health Psychol Rev. 2016;10(4):381–94. 26745243 10.1080/17437199.2015.1138093 PMC5214381

[49] Temple NJ. Front-of-package food labels: a narrative review. Appetite. 2020;144:104485. 31605724 10.1016/j.appet.2019.104485

[50] Ducrot P, Julia C, Méjean C, Kesse-Guyot E, Touvier M, Fezeu LK, et al. Impact of different front-of-pack nutrition labels on consumer purchasing intentions: a randomized controlled trial. Am J Prev Med. 2016;50(5):627–36. 26699246 10.1016/j.amepre.2015.10.020

[51] Hersey JC, Wohlgenant KC, Arsenault JE, Kosa KM, Muth MK. Effects of front-of-package and shelf nutrition labeling systems on consumers. Nutr Rev. 2013;71(1):1–14. 23282247 10.1111/nure.12000

[52] Newman CL, Howlett E, Burton S. Shopper response to front-of-package nutrition labeling programs: potential consumer and retail store benefits. J Retail. 2014;90(1):13–26.

[53] Shea BJ, Reeves BC, Wells G, Thuku M, Hamel C, Moran J, et al. AMSTAR 2: a critical appraisal tool for systematic reviews that include randomised or non-randomised studies of healthcare interventions, or both. BMJ. 2017;358:j4008. 28935701 10.1136/bmj.j4008 PMC5833365

[54] Popay J, Roberts H, Sowden A, Petticrew M, Arai L, Rodgers M, et al. Guidance on the conduct of narrative synthesis in systematic Reviews. A Product from the ESRC Methods Programme. Version 1. 2006. 10.13140/2.1.1018.4643.

[55] Al-Khudairy L, Uthman OA, Walmsley R, Johnson S, Oyebode O. Choice architecture interventions to improve diet and/or dietary behaviour by healthcare staff in high-income countries: a systematic review. BMJ Open. 2019;9(1):023687-NA. 10.1136/bmjopen-2018-023687 PMC6347858 30674487

[56] An R, Shi Y, Shen J, Bullard T, Liu G, Yang Q, et al. Effect of front-of-package nutrition labeling on food purchases: a systematic review. Public Health. 2021;191:59–67. 33517247 10.1016/j.puhe.2020.06.035

[57] Atanasova P, Kusuma D, Pineda E, Frost G, Sassi F, Miraldo M. The impact of the consumer and neighbourhood food environment on dietary intake and obesity-related outcomes: a systematic review of causal impact studies. Soc Sci Med. 2022;299:114879. 35290815 10.1016/j.socscimed.2022.114879 PMC8987734

[58] Bianchi F, Garnett E, Dorsel C, Aveyard P, Jebb SA. Restructuring physical micro-environments to reduce the demand for meat: a systematic review and qualitative comparative analysis. Lancet Planet Health. 2018;2(9):e384–97. 30177007 10.1016/S2542-5196(18)30188-8 PMC6120131

[59] Cameron AJ, Charlton E, Ngan W, Sacks G. A systematic review of the effectiveness of supermarket-based interventions involving product, promotion, or place on the healthiness of consumer purchases. Curr Nutr Rep. 2016;5(3):129–38.

[60] Chu R, Tang T, Hetherington MM. The impact of food packaging on measured food intake: a systematic review of experimental, field and naturalistic studies. Appetite. 2021;166:105579. 34197837 10.1016/j.appet.2021.105579

[61] Funderburk L, Cardaci T, Fink A, Taylor K, Rohde J, Harris D. Healthy behaviors through behavioral design-obesity prevention. Int J Environ Res Public Health. 2020. 10.3390/ijerph17145049. 32674287 10.3390/ijerph17145049 PMC7400269

[62] Golding SE, Bondaronek P, Bunten A, Porter L, Maynard V, Rennie D, et al. Interventions to change purchasing behaviour in supermarkets: a systematic review and intervention content analysis. Health Psychol Rev. 2021;16(2):1–41. 33847250 10.1080/17437199.2021.1911670

[63] Gupta A, Billich N, George NA, Blake MR, Huse O, Backholer K, et al. The effect of front-of-package labels or point-of-sale signage on consumer knowledge, attitudes and behavior regarding sugar-sweetened beverages: a systematic review. Nutr Rev. 2021;79(10):1165–81. 33120419 10.1093/nutrit/nuaa107

[64] Gynell I, Kemps E, Prichard I. The effectiveness of implicit interventions in food menus to promote healthier eating behaviours: a systematic review. Appetite. 2022;173:105997. 35278590 10.1016/j.appet.2022.105997

[65] Hollands GJ, Shemilt I, Marteau TM, Jebb SA, Lewis HB, Wei Y, et al. Portion, package or tableware size for changing selection and consumption of food, alcohol and tobacco. Cochrane Database Syst Rev. 2015(9):CD011045. 10.1002/14651858.CD011045.pub2 PMC4579823 26368271

[66] Hollands GJ, Carter P, Anwer S, King SE, Jebb SA, Ogilvie D, et al. Altering the availability or proximity of food, alcohol, and tobacco products to change their selection and consumption. Cochrane Database Syst Rev. 2019;8:CD012573. 10.1002/14651858.CD012573.pub2 PMC6710643 31452193

[67] Harbers MC, Beulens JWJ, Rutters F, de Boer F, Gillebaart M, Sluijs I, et al. The effects of nudges on purchases, food choice, and energy intake or content of purchases in real-life food purchasing environments: a systematic review and evidence synthesis. Nutr J. 2020;19(1):103. 32943071 10.1186/s12937-020-00623-y PMC7500553

[68] Hartmann-Boyce J, Bianchi F, Piernas C, Riches SP, Frie K, Nourse R, et al. Grocery store interventions to change food purchasing behaviors: a systematic review of randomized controlled trials. Am J Clin Nutr. 2018;107(6):1004–16. 29868912 10.1093/ajcn/nqy045 PMC5985731

[69] Kraak VI, Story M. Influence of food companies’ brand mascots and entertainment companies’ cartoon media characters on children’s diet and health: a systematic review and research needs. Obes Rev. 2015;16(2):107–26. 25516352 10.1111/obr.12237 PMC4359675

[70] Laiou E, Rapti I, Schwarzer R, Fleig L, Cianferotti L, Ngo J, et al. Review: nudge interventions to promote healthy diets and physical activity. Food Policy. 2021;102:102103.

[71] Ljusic N, Fagerstrøm A, Pawar S, Arntzen E. Effects of digitalized front-of-package food labels on healthy food-related behavior: a systematic review. Behav Sci. 2022;12(10):363. 36285932 10.3390/bs12100363 PMC9598805

[72] Marcano-Olivier MI, Horne PJ, Viktor S, Erjavec M. Using nudges to promote healthy food choices in the school dining room: a systematic review of previous investigations. J Sch Health. 2020;90(2):143–57. 31852016 10.1111/josh.12861

[73] Mathur MB, Peacock J, Reichling DB, Nadler J, Bain PA, Gardner CD, et al. Interventions to reduce meat consumption by appealing to animal welfare: meta-analysis and evidence-based recommendations. Appetite. 2021;164:105277. 33984401 10.1016/j.appet.2021.105277 PMC9205607

[74] Meier J, Andor MA, Doebbe FC, Haddaway NR, Reisch LA. Do green defaults reduce meat consumption? Food Policy. 2022;110:102298.

[75] Metcalfe JJ, Ellison B, Hamdi N, Richardson R, Prescott MP. A systematic review of school meal nudge interventions to improve youth food behaviors. Int J Behav Nutr Phys Act. 2020;17(1):77. 32560731 10.1186/s12966-020-00983-y PMC7304192

[76] Osei-Assibey G, Dick S, Macdiarmid JI, Semple S, Reilly JJ, Ellaway A, et al. The influence of the food environment on overweight and obesity in young children: a systematic review. BMJ Open. 2012;2(6):e001538. 23253872 10.1136/bmjopen-2012-001538 PMC3532982

[77] Richardson S, McSweeney L, Spence S. Availability of healthy food and beverages in hospital outlets and interventions in the UK and USA to improve the hospital food environment: a systematic narrative literature review. Nutrients. 2022. 10.3390/nu14081566. 35458128 10.3390/nu14081566 PMC9024949

[78] Shaw S, Ntani G, Baird J, Vogel C. A systematic review of the influences of food store product placement on dietary-related outcomes. Nutr Rev. 2020;78(12):1030–45. 32483615 10.1093/nutrit/nuaa024 PMC7666915

[79] Slapø H, Schjøll A, Strømgren B, Sandaker I, Lekhal S. Efficiency of in-store interventions to impact customers to purchase healthier food and beverage products in real-life grocery stores: a systematic review and meta-analysis. Foods. 2021;10(5):922. 33922185 10.3390/foods10050922 PMC8146080

[80] Stiles G, Collins J, Beck KL. Effectiveness of strategies to decrease animal-sourced protein and/or increase plant-sourced protein in foodservice settings: a systematic literature review. J Acad Nutr Diet. 2022;122(5):1013–48. 34954384 10.1016/j.jand.2021.12.010

[81] Vargas-Alvarez MA, Navas-Carretero S, Palla L, Martínez JA, Almiron-Roig E. Impact of portion control tools on portion size awareness, choice and intake: systematic review and meta-analysis. Nutrients. 2021;13(6):1978. 34207492 10.3390/nu13061978 PMC8229078

[82] Whatnall MC, Patterson AJ, Hutchesson MJ. Effectiveness of nutrition interventions in vending machines to encourage the purchase and consumption of healthier food and drinks in the university setting: a systematic review. Nutrients. 2020. 10.3390/nu12030876. 32213973 10.3390/nu12030876 PMC7146342

[83] Wilson AL, Buckley E, Buckley JD, Bogomolova S. Nudging healthier food and beverage choices through salience and priming. Evidence from a systematic review. Food Qual Prefer. 2016;51:47–64.

[84] Pechey R, Reynolds JP, Cook B, Marteau TM, Jebb SA. Acceptability of policies to reduce consumption of red and processed meat: a population-based survey experiment. J Environ Psychol. 2022;81:101817. 36523649 10.1016/j.jenvp.2022.101817 PMC9742849

[85] Graham F, Barker M, Menon M, Holdsworth M. Acceptability and feasibility of a café-based sustainable food intervention in the UK. Health Promot Int. 2020;35(6):1507–18. 32243498 10.1093/heapro/daaa027

[86] Lombardini C, Lankoski L. Forced choice restriction in promoting sustainable food consumption: intended and unintended effects of the mandatory vegetarian day in Helsinki schools. J Consumer Policy. 2013;36(2):159–78.

[87] Pechey R, Jenkins H, Cartwright E, Marteau TM. Altering the availability of healthier vs. less healthy items in UK hospital vending machines: a multiple treatment reversal design. Int J Behav Nutr Phys Act. 2019;16(1):114. 31775798 10.1186/s12966-019-0883-5 PMC6882209

[88] Nuffield Council on Bioethics. Public health: ethical issues. Nuffield Council on Bioethics; 2007. Report No.: 190438417X.

[89] Kunst JR, Palacios Haugestad CA. The effects of dissociation on willingness to eat meat are moderated by exposure to unprocessed meat: a cross-cultural demonstration. Appetite. 2018;120:356–66. 28951236 10.1016/j.appet.2017.09.016

[90] Mantzari E, Reynolds JP, Jebb SA, Hollands GJ, Pilling MA, Marteau TM. Public support for policies to improve population and planetary health: a population-based online experiment assessing impact of communicating evidence of multiple versus single benefits. Soc Sci Med. 2022;296:114726. 35093794 10.1016/j.socscimed.2022.114726 PMC8907862

[91] Science Advice for Policy by European Academies. Towards Sustainable Food Consumption. Berlin: SAPEA; 2023.

[92] Springmann M, Clark M, Mason-D’Croz D, Wiebe K, Bodirsky BL, Lassaletta L, et al. Options for keeping the food system within environmental limits. Nature. 2018;562(7728):519–25. 30305731 10.1038/s41586-018-0594-0

[93] UK Government. Fit for the future: 10 Year Health Plan for England. 2025. Available from: https://assets.publishing.service.gov.uk/media/6888a0b1a11f859994409147/fit-for-the-future-10-year-health-plan-for-england.pdf.

[94] McKenzie JE, Brennan SE, Ryan RE, Thomson HJ, Johnston RV, Thomas J. Chapter 3: Defining the criteria for including studies and how they will be grouped for the synthesis. In: Higgins JPT, Thomas J, Chandler J, Cumpston M, Li T, Page MJ, et al., editors. Cochrane Handbook for Systematic Reviews of Interventions version 64 (updated August 2023): Cochrane; 2023. Available from: https://www.cochrane.org/authors/handbooks-and-manuals/handbook/current/chapter-03.

[95] Hebert JR, Clemow L, Pbert L, Ockene IS, Ockene JK. Social desirability bias in dietary self-report may compromise the validity of dietary intake measures. Int J Epidemiol. 1995;24(2):389–98. 7635601 10.1093/ije/24.2.389

[96] Buscemi N, Hartling L, Vandermeer B, Tjosvold L, Klassen TP. Single data extraction generated more errors than double data extraction in systematic reviews. J Clin Epidemiol. 2006;59(7):697–703. 16765272 10.1016/j.jclinepi.2005.11.010

[97] Ritchie H. Global inequalities in CO2 emissions: Our World In Data; 2023 [Available from: https://ourworldindata.org/inequality-co2].

[98] Middel CNH, Schuitmaker-Warnaar TJ, Mackenbach JD, Broerse JEW. Systematic review: a systems innovation perspective on barriers and facilitators for the implementation of healthy food-store interventions. Int J Behav Nutr Phys Act. 2019;16(1):1–15. 31752885 10.1186/s12966-019-0867-5 PMC6868845

[99] Vogel C, Dijkstra C, Huitink M, Dhuria P, Poelman MP, Mackenbach JD, et al. Real-life experiments in supermarkets to encourage healthy dietary-related behaviours: opportunities, challenges and lessons learned. Int J Behav Nutr Phys Act. 2023;20(1):73. 37340326 10.1186/s12966-023-01448-8 PMC10280909

[100] Gittelsohn J, Laska MN, Karpyn A, Klingler K, Ayala GX. Lessons learned from small store programs to increase healthy food access. Am J Health Behav. 2014;38(2):307–15. 24629559 10.5993/AJHB.38.2.16 PMC3960288

[101] Foss M, Royston S, Atkinson M, Hawkes C. FRC Food Policy Evidence Review: Engaging with convenience stores for healthier food provision: what works? : Food Research Collaboration; 2019. Available from: https://foodresearch.org.uk/publications/engaging-convenience-stores/.

