# Supplementary material for: Comparing the impact and mechanistic pathways of micro-environmental interventions targeting healthier vs. more environmentally sustainable food options: an overview of reviews
Source: BMC Med. 2025 Oct 24;23:586. doi: 10.1186/s12916-025-04381-8 (PMC12553259; doi:10.1186/s12916-025-04381-8)
Supplement: Supplementary file 1 — Supplementary Material 1. Search strategy. [file 12916_2025_4381_MOESM1_ESM.pdf]

Comparing the impact and mechanistic pathways of micro-environmental interventions targeting healthier vs. more sustainable food options: An overview of reviews

APPENDICES

Contents

APPENDIX A. Search strategy 1

APPENDIX B. Review selection & data extraction.................................. **Error! Bookmark not defined.**

APPENDIX C. Examples of information interventions............................ **Error! Bookmark not defined.**

APPENDIX D. Detailed overview of identified studies .... **Error! Bookmark not defined.**

APPENDIX E. Mediators, moderators and cross-cultural factors. .... **Error! Bookmark not defined.**

APPENDIX A. Search strategy

We searched ASSIA(Proquest)[1987-present], CAB Abstracts(OvidSP)[1973 to 2023 Week 01], Cochrane Database of Systematic Reviews(Cochrane Library, Wiley)[Issue 1 of 12, January 2023), Embase(OvidSP)[1973-present], Medline(OvidSP)[1946-present], PsycINFO(OvidSP)[1806-present] and Science Citation Index, Social Science Citation Index, Conference Proceedings Citation Index - Science & Conference Proceedings Citation Index - Social Science & Humanities(Web of Science Core Collection)[1900-present]. The search strategy was developed in Medline and adapted to other database with the support of Polyglot https://polyglot.sr-accelerator.com/. The search contained title, abstract, author keywords and subject headings, where available, for our main concepts of micro-environmental interventions, healthy food choices, and sustainable food choices. The records were exported to Endnote 20 for storage and transferred to Covidence [44] for deduplication and screening. One review [Liberato SC, Bailie R, Brimblecombe J. Nutrition interventions at point-of-sale to encourage healthier food purchasing: a systematic review. BMC Public Health.

2014;14(1):919] was excluded after the forward and backward search due to containing only one study that overlapped with a meta-analysis in another included review [66].

Search strategies: 2023

	Medline (Ovid MEDLINE® Epub Ahead of Print, In-Process & Other Non-Indexed Citations, Ovid MEDLINE® Daily and Ovid MEDLINE®) 1946 to present	
1	((microenvironment* or micro-environment* or choice architecture) adj3 intervention?).ti,ab,kf.	91
2	Food Packaging/ or edible films/	6271
3	food labeling/	4464
4	(ecolabel* or eco-label* or sustainab* label*).ti,ab,kf.	116
5	((food or nutrition* or nutrient* or diet* or menu or drink? or beverage?) adj2 label*).ti,ab,kf.	4595
6	((food or nutrition* or nutrient* or diet* or menu or drink? or beverage?) adj2 packag*).ti,ab,kf.	5579
7	((food or nutrition* or nutrient* or diet* or menu or drink? or beverage?) adj2 (marketing or promotion* or advert* or nudg*)).ti,ab,kf.	2609
8	(("front of pack*" or fop or "point of sale" or pos) adj3 label*).ti,ab,kf.	614
9	supermarkets/ and (Marketing/ or exp Health Promotion/ or Health Education/)	24
10	(Restaurants/ or food services/) and (Marketing/ or exp Health Promotion/ or Health Education/)	1256
11	(commerce/ or small business/) and (Marketing/ or exp Health Promotion/ or Health Education/)	1666
12	(food environment and (marketing or promotion* or advert* or nudg*)).ti,ab,kf.	456
13	(((sale? or selling or purchas*) adj3 point?) and (marketing or promotion* or advert* or nudg*)).ti,ab,kf.	509
14	((retailer? or supermarket? or market? or shop? or store?) and (marketing or promotion* or advert* or nudg*)).ti,ab,kf.	8836
15	((restaurant? or bistro? or cafe? or pub? or public house? or tavern? or inn?) and (marketing or promotion* or advert* or nudg*)).ti,ab,kf.	659
16	Workplace/ and (Marketing/ or exp Health Promotion/ or Health Education/)	2676
17	Schools/ and (Marketing/ or exp Health Promotion/ or Health Education/)	5275

18 ((cafeteria? or canteen? or ((dining or dinner or lunch) adj2 (room? 301 or hall?))) and (marketing or promotion* or advert* or nudg*)).ti,ab,kf.

19 ((work or workplace? or school? or college?) and (marketing or 27502 promotion* or advert* or nudg*)).ti,ab,kf.

20 or/2-19 60983

21 exp "Conservation of Natural Resources"/ 110761

22 carbon footprint/ or ecosystem/ or exp biodiversity/ 212893

23 Greenhouse Gases/ 2099

24 exp air pollutants/ or exp fossil fuels/ 129060

25 Environment/ 67123

26 exp climate change/ or greenhouse effect/ 33833

27 exp soil pollutants/ 48917

28 waste water/ or exp water pollutants/ or water supply/ 185191

29 exp Forests/ 15329

30 sustainab*.ti,ab,kf. 123082

31 **environment*.ti,ab,kf.** 1281930

32 (carbon adj2 (footprint or exchange or emission*)).ti,ab,kf. 8875

33 (air adj2 pollut*).ti,ab,kf. 44317 34 (greenhouse gas* or greenhouse effect* or ghg or climat* change or 77450 global warming or fossil fuel*).ti,ab,kf.

35 (acidification or eurtofication).ti,ab,kf. 24747

36 (ecosystem* or biodivers* or bio-divers* or (natural adj2 (resource* 154163 or environment*))).ti,ab,kf.

37 ("land use" or land usage or (land adj2 clear*) or forest* or 123021 rainforest* or deforest* or (soil adj2 pollut*)).ti,ab,kf.

38 ("water use" or "water usage" or (water adj2 pollut*)).ti,ab,kf. 18492

39 (life cycle? or lifecycle? or life span? or lifespan?).ti,ab,kf. 115680

40 or/21-39 2059464

41 exp eating/ or feeding behavior/ or carnivory/ or food preferences/ 179655 or herbivory/

42 diet/ or diet, vegan/ or diet, healthy/ or portion size/ or serving size/ 188162 43 Choice Behavior/ 34706

44 consumer behavior/ or health behavior/ 79040

45 exp Overweight/pc 22660

46 ((food or nutrition* or nutrient* or diet* or menu) adj2 (choice? or 163963 purchas* or intake or consum* or chang*)).ti,ab,kf.

47 ((portion? or plate?) adj2 (size or serving?)).ti,ab,kf. 1739

48 ((food or nutrition* or nutrient* or diet* or menu) adj2 (attitude? or 5282
perception? or perspective? or view* or intention?)).ti,ab,kf.

49 (sustain* adj2 (choice? or purchas* or intake or consum* or 5160
chang*)).ti,ab,kf.

50 (sustain* adj2 (attitude? or perception? or perspective? or view* or 912
intention?)).ti,ab,kf.

51 ((purchas* or buy*) adj2 (choice? or chang* or behav*)).ti,ab,kf. 1501

52 ((purchas* or buy*) adj2 (attitude? or perception? or perspective? 1133 or view*
or intention?)).ti,ab,kf.

53 ((meat or fat? or sugar? or fruit or vegetable? or dairy) adj2 (choice? 46288 or
purchas* or intake or consum* or chang*)).ti,ab,kf.

54 ((meat or fat? or sugar? or dairy) adj2 (reduc* or decreas* or declin* 26275 or
replac*)).ti,ab,kf.

55 ((fruit or vegetable) adj2 increas*).ti,ab,kf. 3373

56 (meat adj (alternative? or substitute? or replacement? or 468
analogue?)).ti,ab,kf.

57 ((plant-based or plant-sourced or dairy free or vegan) adj2 (product? 1149 or
protein? or drink? or milk? or yoghurt? or cheese? or butter or margarine or
spread)).ti,ab,kf.

58 ((soya or oat or almond or coconut or rice or pea) adj2 (product? or 5085 protein?
or drink? or beverage? or milk? or yoghurt? or cheese? or butter or margarine or
spread)).ti,ab,kf.

59 (health* adj2 (eating or food? or choice? or behav? or 30048 purchas*)).ti,ab,kf.

60 ((obes* or overweight or weight gain or weight increas* or increas* 19609 weight)
adj5 prevent*).ti,ab,kf.

61 or/41-60 621623

62 20 and 40 and 61 3274

63 1 or 62 3361

64 (systematic review or meta-analysis or review).pt. 3255009

65 (search* or meta analysis).ti,ab. or meta analysis/ 720507

66 (scoping review or (evidence adj2 (review or synthesis or 51729
map*))).ti,ab,kf.

67 64 or 65 or 66 3579589

68 63 and 67 669 Embase 1974 to present

1 ((microenvironment* or micro-environment* or choice 108
architecture) adj3 intervention?).ti,ab,kf.

2 exp *food packaging/ 5670

3 (ecolabel* or eco-label* or sustainab* label*).ti,ab,kf. 173

4 ((food or nutrition* or nutrient* or diet* or menu or drink? or 5804 beverage?) adj2 label*).ti,ab,kf.

5 ((food or nutrition* or nutrient* or diet* or menu or drink? or 5369 beverage?) adj2 packag*).ti,ab,kf.

6 ((food or nutrition* or nutrient* or diet* or menu or drink? or 3127 beverage?) adj2 (marketing or promotion* or advert* or nudg*)).ti,ab,kf.

7 (("front of pack*" or fop or "point of sale" or pos) adj3 708 label*).ti,ab,kf.

8 (retail outlet/ or exp grocery store/) and (marketing/ or health 129 education/ or exp health promotion/ or nutrition education/)

9 (home delivered meal/ or restaurant/ or "takeaway (food)"/) 108 and (marketing/ or health education/ or exp health promotion/ or nutrition education/)

10 commercial phenomena/ and (marketing/ or health education/ 2810 or exp health promotion/ or nutrition education/)

11 (food environment and (marketing or promotion* or advert* or 553 nudg*)).ti,ab,kf.

12 (((sale? or selling or purchas*) adj3 point?) and (marketing or 596 promotion* or advert* or nudg*)).ti,ab,kf.

13 ((retailer? or supermarket? or market? or shop? or store?) and 11839 (marketing or promotion* or advert* or nudg*)).ti,ab,kf.

14 ((restaurant? or bistro? or cafe? or pub? or public house? or 875 tavern? or inn?) and (marketing or promotion* or advert* or nudg*)).ti,ab,kf.

15 Workplace/ and (marketing/ or health education/ or exp health 3931 promotion/ or nutrition education/)

16 (school/ or college/ or community college/ or high school/ or 9176 middle school/ or primary school/) and (marketing/ or health education/ or exp health promotion/ or nutrition education/)

17 ((cafeteria? or canteen? or ((dining or dinner or lunch) adj2 403 (room? or hall?))) and (marketing or promotion* or advert* or nudg*)).ti,ab,kf.

18 ((work or workplace? or school? or college?) and (marketing or 34925 promotion* or advert* or nudg*)).ti,ab,kf.

19 or/2-18 72288

20 exp *environmental protection/ 39646

21 exp *environmental footprint/ or *ecosystem/ or exp 136197 *biodiversity/

22 exp *air pollutant/ 43603 23 *fossil fuel/ 1481

24 *Environment/ 22290

25 **exp *climate change/ or *environmental impact/ or** 36617

***greenhouse effect/ or *"human impact (environment)"/**

26 *soil pollutant/ 7291

27 exp *wastewater/ or exp *water pollutant/ or *water supply/ 43167

28 exp *forest/ 14070

29 sustainab*.ti,ab,kf. 134148

30 environment*.ti,ab,kf. 1480170

31 (carbon adj2 (footprint or exchange or emission*)).ti,ab,kf. 9357

32 (air adj2 pollut*).ti,ab,kf. 56203 33 (greenhouse gas* or greenhouse effect* or ghg or climat* 78280 change or global warming or fossil fuel*).ti,ab,kf.

34 (acidification or eurtofication).ti,ab,kf. 29498

35 (ecosystem* or biodivers* or bio-divers* or (natural adj2 156222 (resource* or environment*))).ti,ab,kf.

36 ("land use" or land usage or (land adj2 clear*) or forest* or 134605 rainforest* or deforest* or (soil adj2 pollut*)).ti,ab,kf.

37 ("water use" or "water usage" or (water adj2 pollut*)).ti,ab,kf. 20508

38 (life cycle? or lifecycle? or life span? or lifespan?).ti,ab,kf. 135559

39 or/20-38 2121212

40 exp *food intake/ 116359

41 exp *feeding behavior/ 71240

42 *diet/ or *healthy diet/ or exp *vegetarian diet/ 77819

43 *consumer attitude/ or *health behavior/ 30201

44 exp *obesity/pc 11060

45 ((food or nutrition* or nutrient* or diet* or menu) adj2 210651 (choice? or purchas* or intake or consum* or chang*)).ti,ab,kf.

46 ((portion? or plate?) adj2 (size or serving?)).ti,ab,kf. 2310

47 ((food or nutrition* or nutrient* or diet* or menu) adj2 6525 (attitude? or perception? or perspective? or view* or intention?)).ti,ab,kf.

48 (sustain* adj2 (choice? or purchas* or intake or consum* or 6576 chang*)).ti,ab,kf.

49 (sustain* adj2 (attitude? or perception? or perspective? or 934 view* or intention?)).ti,ab,kf.

50 ((purchas* or buy*) adj2 (choice? or chang* or 1732 behav*)).ti,ab,kf.

51 ((purchas* or buy*) adj2 (attitude? or perception? or 1007 perspective? or view* or intention?)).ti,ab,kf.

52 ((meat or fat? or sugar? or fruit or vegetable? or dairy) adj2 57859 (choice? or purchas* or intake or consum* or chang*)).ti,ab,kf.

53 ((meat or fat? or sugar? or dairy) adj2 (reduc* or decreas* or 33242 declin* or replac*)).ti,ab,kf.

54 ((fruit or vegetable) adj2 increas*).ti,ab,kf. 3664

55 (meat adj (alternative? or substitute? or replacement? or 445 analogue?)).ti,ab,kf.

56 ((plant-based or plant-sourced or dairy free or vegan) adj2 1213 (product? or protein? or drink? or milk? or yoghurt? or cheese? or butter or margarine or spread)).ti,ab,kf.

57 ((soya or oat or almond or coconut or rice or pea) adj2 5380 (product? or protein? or drink? or beverage? or milk? or yoghurt? or cheese? or butter or margarine or spread)).ti,ab,kf.

58 (health* adj2 (eating or food? or choice? or behav? or 37423 purchas*)).ti,ab,kf.

59 ((obes* or overweight or weight gain or weight increas* or 26468 increas* weight) adj5 prevent*).ti,ab,kf.

60 or/40-59 562880

61 19 and 39 and 60 3191

62 1 or 61 3296

63 systematic review/ or exp meta analysis/ or review/ 3130258

64 (search* or meta analysis).ti,ab. or review.pt. 3626468

65 (scoping review or (evidence adj2 (review or synthesis or 56507 map*))).ti,ab,kf.

66 63 or 64 or 65 3818281

67 62 and 66 560

CAB Abstracts <1973 to 2023 Week 01>

1 ((microenvironment* or micro-environment* or choice 27 architecture) adj3 intervention?).ti,ab,hw.

2 labelling/ or nutrition labelling/ or food packaging/ 24579

3 (ecolabel* or eco-label* or sustainab* label*).ti,ab,hw. 880

4 ((food or nutrition* or nutrient* or diet* or menu or drink? or 6295 beverage?) adj2 label*).ti,ab,hw.

5 ((food or nutrition* or nutrient* or diet* or menu or drink? or 15115 beverage?) adj2 packag*).ti,ab,hw.

6 ((food or nutrition* or nutrient* or diet* or menu or drink? or 11059 beverage?) adj2 (marketing or promotion* or advert* or nudg*)).ti,ab,hw.

7 (("front of pack*" or fop or "point of sale" or pos) adj3 533 label*).ti,ab,hw.

8 (food environment and (marketing or promotion* or advert* or 500 nudg*)).ti,ab,hw.

9 (((sale? or selling or purchas*) adj3 point?) and (marketing or 783 promotion* or advert* or nudg*)).ti,ab,hw.

10 ((retailer? or supermarket? or market? or shop? or store?) and 63162 (marketing or promotion* or advert* or nudg*)).ti,ab,hw.

11 ((restaurant? or bistro? or cafe? or pub? or public house? or 2685 tavern? or inn?) and (marketing or promotion* or advert* or nudg*)).ti,ab,hw.

12 ((cafeteria? or canteen? or ((dining or dinner or lunch) adj2 376 (room? or hall?))) and (marketing or promotion* or advert* or nudg*)).ti,ab,hw.

13 ((work or workplace? or school? or college?) and (marketing or 20248 promotion* or advert* or nudg*)).ti,ab,hw.

14 (markets/ or exp shops/) and (exp marketing/ or health 6160 promotion/ or health education/)

15 exp dining facilities/ and (exp marketing/ or health promotion/ 1012 or health education/)

16 small businesses/ and (exp marketing/ or health promotion/ or 569 health education/)

17 work places/ and (exp marketing/ or health promotion/ or 412 health education/)

18 (schools/ or elementary schools/ or high schools/) and (exp 1832 marketing/ or health promotion/ or health education/)

19 or/2-18 113387

20 environment/ 52944

21 environmental impact/ or carbon footprint/ or life cycle 81032 assessment/

22 exp climate change/ or greenhouse effect/ 155954

23 air pollutants/ or greenhouse gases/ 51338

24 environmental protection/ 28565 25 resource conservation/ or nature conservation/ or soil 71712 conservation/ or water conservation/

26 pollution/ or air pollution/ or soil pollution/ or water pollution/ 352574

27 sustainability/ or "sustainable land use"/ 129588

28 watewater/ or water quality/ 192467

29 exp fossil fuels/ 4622

30 exp forests/ 295555

31 sustainab*.ti,ab,hw. 275996

32 environment*.ti,ab,hw. 1E+06

33 (carbon adj2 (footprint or exchange or emission*)).ti,ab,hw. 14912

34 (air adj2 pollut*).ti,ab,hw. 69276

35 (greenhouse gas* or greenhouse effect* or ghg or climat* 213522 change or global warming or fossil fuel*).ti,ab,hw.

36 (acidification or eurtofication).ti,ab,hw. 23558

37 (ecosystem* or biodivers* or bio-divers* or (natural adj2 399329 (resource* or environment*))).ti,ab,hw.

38 ("land use" or land usage or (land adj2 clear*) or forest* or 843399 rainforest* or deforest* or (soil adj2 pollut*)).ti,ab,hw.

39 ("water use" or "water usage" or (water adj2 pollut*)).ti,ab,hw. 207456

40 (life cycle? or lifecycle? or life span? or lifespan?).ti,ab,hw. 93696

41 or/20-40 2E+06

42 food intake/ or food consumption/ or feeding behaviour/ or exp 133541 eating patterns/ or exp food preferences/ or feeding habits/

43 diets/ or vegetarian diets/ or vegans/ or herbivory/ or 233973 vegetarianism/

44 exp plant milks/ or exp simulated foods/ or exp soyabean 49603 products/

45 exp consumer behaviour/ or consumer attitudes/ 31714

46 health behaviour/ 8896

47 ((food or nutrition* or nutrient* or diet* or menu) adj2 (choice? 188793 or purchas* or intake or consum* or chang*)).ti,ab,hw.

48 ((portion? or plate?) adj2 (size or serving?)).ti,ab,hw. 1893

49 ((food or nutrition* or nutrient* or diet* or menu) adj2 9061 (attitude? or perception? or perspective? or view* or intention?)).ti,ab,hw.

50 (sustain* adj2 (choice? or purchas* or intake or consum* or 4024 chang*)).ti,ab,hw.

51 (sustain* adj2 (attitude? or perception? or perspective? or 2378 view* or intention?)).ti,ab,hw.

52 ((purchas* or buy*) adj2 (choice? or chang* or 3176 behav*)).ti,ab,hw.

53 ((purchas* or buy*) adj2 (attitude? or perception? or 3068 perspective? or view* or intention?)).ti,ab,hw.

54 ((meat or fat? or sugar? or fruit or vegetable? or dairy) adj2 57816 (choice? or purchas* or intake or consum* or chang*)).ti,ab,hw.

55 ((meat or fat? or sugar? or dairy) adj2 (reduc* or decreas* or 48766 declin* or replac*)).ti,ab,hw.

56 ((fruit or vegetable) adj2 increas*).ti,ab,hw. 14810

57 (meat adj (alternative? or substitute? or replacement? or 1102
analogue?)).ti,ab,hw.

58 ((plant-based or plant-sourced or dairy free or vegan) adj2 1617 (product? or
protein? or drink? or milk? or yoghurt? or cheese?
or butter or margarine or spread)).ti,ab,hw.

59 ((soya or oat or almond or coconut or rice or pea) adj2 (product? 25807 or
protein? or drink? or beverage? or milk? or yoghurt? or cheese? or butter or
margarine or spread)).ti,ab,hw.

60 (health* adj2 (eating or food? or choice? or behav? or 29779 purchas*)).ti,ab,hw.

61 ((obes* or overweight or weight gain or weight increas* or 12375 increas*
weight) adj5 prevent*).ti,ab,hw.

62 or/42-61 611066

63 19 and 41 and 62 5093

64 1 or 63 5117

65 reviews/ or literature reviews/ or scoping reviews/ or systematic 412364 reviews/ or
meta analysis/

66 (scoping review or (evidence adj2 (review or synthesis or 6421
map*))).ti,ab,hw.

67 65 or 66 414418

68 64 and 67 471 PsycINFO 1806 to present

1 ((microenvironment* or micro-environment* or choice 38
architecture) adj3 intervention?).ti,ab,hw.

2 (ecolabel* or eco-label* or sustainab* label*).ti,ab,hw. 104

3 ((food or nutrition* or nutrient* or diet* or menu or drink? or 935
beverage?) adj2 label*).ti,ab,hw.

4 ((food or nutrition* or nutrient* or diet* or menu or drink? or 412
beverage?) adj2 packag*).ti,ab,hw.

5 ((food or nutrition* or nutrient* or diet* or menu or drink? or 1093
beverage?) adj2 (marketing or promotion* or advert* or nudg*)).ti,ab,hw.

6 (("front of pack*" or fop or "point of sale" or pos) adj3 140 label*).ti,ab,hw.

7 (food environment and (marketing or promotion* or advert* or 166
nudg*)).ti,ab,hw.

8 (((sale? or selling or purchas*) adj3 point?) and (marketing or 423 promotion* or
advert* or nudg*)).ti,ab,hw.

9 ((retailer? or supermarket? or market? or shop? or store?) and 15134
(marketing or promotion* or advert* or nudg*)).ti,ab,hw.

10 ((restaurant? or bistro? or cafe? or pub? or public house? or 843 tavern? or inn?) and (marketing or promotion* or advert* or nudg*)).ti,ab,hw.

11 ((cafeteria? or canteen? or ((dining or dinner or lunch) adj2 160 (room? or hall?))) and (marketing or promotion* or advert* or nudg*)).ti,ab,hw.

12 ((work or workplace? or school? or college?) and (marketing or 27475 promotion* or advert* or nudg*)).ti,ab,hw.

13 or/2-12 42887

14 exp "nature (environment)"/ or environment/ 22517

15 environmental effects/ 10210

16 exp climate change/ 3245

17 pollution/ 1958

18 environmental attitudes/ or "conservation (ecological 8644 behavior)"/

19 sustainab*.ti,ab,hw. 26135

20 environment*.ti,ab,hw. 430511

21 (carbon adj2 (footprint or exchange or emission*)).ti,ab,hw. 372

22 (air adj2 pollut*).ti,ab,hw. 1383 23 (greenhouse gas* or greenhouse effect* or ghg or climat* 5439 change or global warming or fossil fuel*).ti,ab,hw.

24 (acidification or eurtofication).ti,ab,hw. 341

25 (ecosystem* or biodivers* or bio-divers* or (natural adj2 11209 (resource* or environment*))).ti,ab,hw.

26 ("land use" or land usage or (land adj2 clear*) or forest* or 8539 rainforest* or deforest* or (soil adj2 pollut*)).ti,ab,hw.

27 ("water use" or "water usage" or (water adj2 pollut*)).ti,ab,hw. 269

28 (life cycle? or lifecycle? or life span? or lifespan?).ti,ab,hw. 34974

29 or/14-28 490643

30 food intake/ or food preferences/ or eating attitudes/ 21932

31 diets/ 14945

32 exp eating behavior/ or health behavior/ 55230

33 consumer behavior/ or consumer attitudes/ 42634

34 choice behavior/ 20340 35 ((food or nutrition* or nutrient* or diet* or menu) adj2 (choice? 33654 or purchas* or intake or consum* or chang*)).ti,ab,hw.

36 ((portion? or plate?) adj2 (size or serving?)).ti,ab,hw. 462

37 ((food or nutrition* or nutrient* or diet* or menu) adj2 2291 (attitude? or perception? or perspective? or view* or intention?)).ti,ab,hw.

38 (sustain* adj2 (choice? or purchas* or intake or consum* or 2914
chang*)).ti,ab,hw.

39 (sustain* adj2 (attitude? or perception? or perspective? or 691 view* or
intention?)).ti,ab,hw.

40 ((purchas* or buy*) adj2 (choice? or chang* or 3045 behav*)).ti,ab,hw.

41 ((purchas* or buy*) adj2 (attitude? or perception? or 3871 perspective? or view*
or intention?)).ti,ab,hw.

42 ((meat or fat? or sugar? or fruit or vegetable? or dairy) adj2 6489 (choice? or
purchas* or intake or consum* or chang*)).ti,ab,hw.

43 ((meat or fat? or sugar? or dairy) adj2 (reduc* or decreas* or 1397 declin* or
replac*)).ti,ab,hw.

44 ((fruit or vegetable) adj2 increas*).ti,ab,hw. 729

45 (meat adj (alternative? or substitute? or replacement? or 61
analogue?)).ti,ab,hw.

46 ((plant-based or plant-sourced or dairy free or vegan) adj2 41 (product? or
protein? or drink? or milk? or yoghurt? or cheese?
or butter or margarine or spread)).ti,ab,hw.

47 ((soya or oat or almond or coconut or rice or pea) adj2 39
(product? or protein? or drink? or beverage? or milk? or yoghurt?
or cheese? or butter or margarine or spread)).ti,ab,hw.

48 (health* adj2 (eating or food? or choice? or behav? or 9414 purchas*)).ti,ab,hw.

49 ((obes* or overweight or weight gain or weight increas* or 4629 increas* weight)
adj5 prevent*).ti,ab,hw.

50 or/30-49 163277

51 13 and 29 and 50 2321

52 1 or 51 2355

53 systematic review/ or exp "literature review"/ or meta analysis/ 28666

54 (search* or meta analysis).ti,ab. or (meta analysis or 159621 metasynthesis or
"systematic review").md.

55 (scoping review or (evidence adj2 (review or synthesis or 12659
map*))).ti,ab,hw.

56 53 or 54 or 55 189427

57 52 and 56 132

Cochrane Library

ID	Search	Hits
#1	((microenvironment*:ti,ab,kw OR micro-environment*:ti,ab,kw OR "choice architecture":ti,ab,kw) NEAR/3 intervention?:ti,ab,kw)	16
#2	[mh ^"Food Packaging"] OR [mh ^"edible films"]	36
#3	ecolabel*:ti,ab,kw OR eco-label*:ti,ab,kw OR (sustainab* NEXT label*):ti,ab,kw	8
#4	((food:ti,ab,kw OR nutrition*:ti,ab,kw OR nutrient*:ti,ab,kw OR diet*:ti,ab,kw OR menu:ti,ab,kw OR drink?:ti,ab,kw OR beverage?:ti,ab,kw) NEAR/2 label*):ti,ab,kw	761
#5	((food:ti,ab,kw OR nutrition*:ti,ab,kw OR nutrient*:ti,ab,kw OR diet*:ti,ab,kw OR menu:ti,ab,kw OR drink?:ti,ab,kw OR beverage?:ti,ab,kw) NEAR/2 packag*:ti,ab,kw)	341
#6	((food:ti,ab,kw OR nutrition*:ti,ab,kw OR nutrient*:ti,ab,kw OR diet*:ti,ab,kw OR menu:ti,ab,kw OR drink?:ti,ab,kw OR beverage?:ti,ab,kw) NEAR/2 (marketing:ti,ab,kw OR promotion*:ti,ab,kw OR advert*:ti,ab,kw OR nudg*:ti,ab,kw))	353
#7	((("front of" NEXT pack*):ti,ab,kw OR fop:ti,ab,kw OR "point of sale":ti,ab,kw OR pos:ti,ab,kw) NEAR/3 label*:ti,ab,kw)	131
#8	[mh ^supermarkets] AND ([mh ^Marketing] OR [mh "Health Promotion"] OR [mh ^"Health Education"])	2
#9	([mh ^Restaurants] OR [mh ^"food services"]) AND ([mh ^Marketing] OR [mh "Health Promotion"] OR [mh ^"Health Education"])	125
#10	([mh ^commerce] OR [mh ^"small business"]) AND ([mh ^Marketing] OR [mh "Health Promotion"] OR [mh ^"Health Education"])	66
#11	("food environment":ti,ab,kw AND (marketing:ti,ab,kw OR promotion*:ti,ab,kw OR advert*:ti,ab,kw OR nudg*:ti,ab,kw)) OR (((sale?:ti,ab,kw OR selling:ti,ab,kw OR purchas*:ti,ab,kw) NEAR/3 point?:ti,ab,kw) AND (marketing:ti,ab,kw OR promotion*:ti,ab,kw OR advert*:ti,ab,kw OR nudg*:ti,ab,kw))	97
#12	((retailer?:ti,ab,kw OR supermarket?:ti,ab,kw OR market?:ti,ab,kw OR shop?:ti,ab,kw OR store?:ti,ab,kw) AND (marketing:ti,ab,kw OR promotion*:ti,ab,kw OR advert*:ti,ab,kw OR nudg*:ti,ab,kw))	757

#13	((restaurant?:ti,ab,kw OR bistro?:ti,ab,kw OR cafe?:ti,ab,kw OR pub?:ti,ab,kw OR ("public" NEXT house?):ti,ab,kw OR tavern?:ti,ab,kw OR inn?:ti,ab,kw) AND (marketing:ti,ab,kw OR promotion*:ti,ab,kw OR advert*:ti,ab,kw OR nudg*:ti,ab,kw))	234
#14	[mh ^Workplace] AND ([mh ^Marketing] OR [mh "Health Promotion"] OR [mh ^"Health Education"])	382
#15	[mh ^Schools] AND ([mh ^Marketing] OR [mh "Health Promotion"] OR [mh ^"Health Education"])	743
#16	((cafeteria?:ti,ab,kw OR canteen?:ti,ab,kw OR ((dining:ti,ab,kw OR dinner:ti,ab,kw OR lunch:ti,ab,kw) NEAR/2 (room?:ti,ab,kw OR hall?:ti,ab,kw))) AND (marketing:ti,ab,kw OR promotion*:ti,ab,kw OR advert*:ti,ab,kw OR nudg*:ti,ab,kw))	111
#17	((work:ti,ab,kw OR workplace?:ti,ab,kw OR school?:ti,ab,kw OR college?:ti,ab,kw) AND (marketing:ti,ab,kw OR promotion*:ti,ab,kw OR advert*:ti,ab,kw OR nudg*:ti,ab,kw))	5298
#18	#1 OR #2 OR #3 OR #4 OR #5 OR #6 OR #7 OR #8 OR #9 OR #10 OR #11 OR #12 OR #13 OR #14 OR #15 OR #16 OR #17 in Cochrane Reviews	125

Web of Science Core Collection

#	Search Query	Results
1	TS=((microenvironment* OR micro-environment* OR "choice NEAR/3 intervention$)	139 architecture")
2	TS=(ecolabel* OR eco-label* OR "sustainab* label*") OR TS=((food OR nutrition* OR nutrient* OR diet* OR menu OR drink$ OR beverage$) NEAR/2 label*) OR TS=((food OR nutrition* OR nutrient* OR diet* OR menu OR drink$ OR beverage$) NEAR/2 packag*) OR TS=((food OR nutrition* OR nutrient* OR diet* OR menu OR drink$ OR beverage$) NEAR/2 (marketing OR promotion* OR advert* OR nudg*)) OR TS=(("front of pack*" OR fop OR "point of sale" OR pos) NEAR/3 label*) OR TS=("food environment" AND (marketing OR promotion* OR advert* OR nudg*)) OR TS=(((sale$ OR selling OR purchas*) NEAR/3 point$) AND (marketing OR promotion* OR advert* OR nudg*)) OR TS=((retailer$ OR supermarket$ OR market$ OR shop$ OR store$) AND (marketing OR promotion* OR advert* OR nudg*)) OR TS=((restaurant$ OR bistro$ OR cafe$ OR pub$ OR "public house$" OR tavern$ OR inn$) AND (marketing OR promotion* OR advert* OR nudg*)) OR TS=((cafeteria$ OR canteen$ OR ((dining OR dinner OR lunch) NEAR/2 (room$ OR hall$))) AND (marketing OR promotion* OR advert* OR nudg*)) OR TS=((work OR workplace$ OR school$ OR college$) AND (marketing OR promotion* OR advert* OR nudg*))	706018
3	TS=(carbon NEAR/2 (footprint OR exchange OR emission*)) OR TS=(air NEAR/2 pollut*) OR TS=("greenhouse gas*" OR "greenhouse effect*" OR ghg OR "climat* change" OR "global warming" OR "fossil fuel*") OR TS=(acidification OR eurtofication) OR TS=(ecosystem* OR biodivers* OR bio-divers* OR (natural NEAR/2 (resource* OR environment*))) OR TS=("land use" OR "land usage" OR (land NEAR/2 clear*) OR forest* OR rainforest* OR deforest* OR (soil NEAR/2 pollut*)) OR TS=("water use" OR "water usage" OR (water NEAR/2 pollut*)) OR TS=("life cycle$" OR lifecycle$ OR "life span$" OR lifespan$)	1686374
4	TS=((food OR nutrition* OR nutrient* OR diet* OR menu) NEAR/2 (choice$ OR purchas* OR intake OR consum* OR chang*)) OR TS=((portion$ OR plate$) NEAR/2 (size OR serving$)) OR TS=((food	453569

	OR nutrition* OR nutrient* OR diet* OR menu) NEAR/2 (attitude$ OR perception$ OR perspective$ OR view* OR intention$)) OR TS=(sustain* NEAR/2 (choice$ OR purchas* OR intake OR consum* OR chang*)) OR TS=(sustain* NEAR/2 (attitude$ OR perception$ OR perspective$ OR view* OR intention$)) OR TS=((purchas* OR buy*) NEAR/2 (choice$ OR chang* OR behav*)) OR TS=((purchas* OR buy*) NEAR/2 (attitude$ OR perception$ OR perspective$ OR view* OR intention$)) OR TS=((meat OR fat$ OR sugar$ OR fruit OR vegetable$ OR dairy) NEAR/2 (choice$ OR purchas* OR intake OR consum* OR chang*)) OR TS=((meat OR fat$ OR sugar$ OR dairy) NEAR/2 (reduc* OR decreas* OR declin* OR replac*)) OR TS=((fruit OR vegetable) NEAR/2 increas*) OR TS=(meat NEAR/0 (alternative$ OR substitute$ OR replacement$ OR analogue$)) OR TS=((plantbased OR plant-sourced OR "dairy free" OR vegan) NEAR/2 (product$ OR protein$ OR drink$ OR milk$ OR yoghurt$ OR cheese$ OR butter OR margarine OR spread)) OR TS=((soya OR oat OR almond OR coconut OR rice OR pea) NEAR/2 (product$ OR protein$ OR drink$ OR beverage$ OR milk$ OR yoghurt$ OR cheese$ OR butter OR margarine OR spread)) OR TS=(health* NEAR/2 (eating OR food$ OR choice$ OR behav$ OR purchas*)) OR TS=((obes* OR overweight OR "weight gain" OR "weight increas*" OR "increas* weight") NEAR/5 prevent*)	
5	#2 AND #3 AND #4	2324
6	#1 OR #5	2463
7	TS=("systematic review" OR meta-analysis) OR TI=review OR TS=("scoping review" OR (evidence NEAR/2 (review OR synthesis OR map*)))	1017196
8	#6 AND #7	181
9	(#1 OR #5) AND (DT==("REVIEW"))	343
10	#8 OR #9	374

ASSIA:

Set#: S1	Searched for: ((TI,AB,IF(microenvironment*) OR TI,AB,IF(micro-environment*) OR TI,AB,IF("choice architecture")) NEAR/3 TI,AB,IF(intervention?))	12
Set#: S2	Searched for: (TI,AB,IF(ecolabel*) OR TI,AB,IF(eco-label*) OR TI,AB,IF("sustainab* label*"))	23
Set#: S3	Searched for: ((TI,AB,IF(food) OR TI,AB,IF(nutrition*) OR TI,AB,IF(nutrient*) OR TI,AB,IF(diet*) OR TI,AB,IF(menu) OR TI,AB,IF(drink?) OR TI,AB,IF(beverage?)) NEAR/2 TI,AB,IF(label*))	376
Set#: S4	Searched for: ((TI,AB,IF(food) OR TI,AB,IF(nutrition*) OR TI,AB,IF(nutrient*) OR TI,AB,IF(diet*) OR TI,AB,IF(menu) OR TI,AB,IF(drink?) OR TI,AB,IF(beverage?)) NEAR/2 TI,AB,IF(packag*))	134
Set#: S5	Searched for: ((TI,AB,IF(food) OR TI,AB,IF(nutrition*) OR TI,AB,IF(nutrient*) OR TI,AB,IF(diet*) OR TI,AB,IF(menu) OR TI,AB,IF(drink?) OR TI,AB,IF(beverage?)) NEAR/2 (TI,AB,IF(marketing) OR TI,AB,IF(promotion*) OR TI,AB,IF(advert*) OR TI,AB,IF(nudg*)))	535
Set#: S6	Searched for: ((TI,AB,IF("front of pack*") OR TI,AB,IF(fop) OR TI,AB,IF("point of sale") OR TI,AB,IF(pos)) NEAR/3 TI,AB,IF(label*))	28
Set#: S7	Searched for: (TI,AB,IF("food environment") AND (TI,AB,IF(marketing) OR TI,AB,IF(promotion*) OR TI,AB,IF(advert*) OR TI,AB,IF(nudg*)))	54
Set#: S8	Searched for: (((TI,AB,IF(sale?) OR TI,AB,IF(selling) OR TI,AB,IF(purchas*)) NEAR/3 TI,AB,IF(point?)) AND (TI,AB,IF(marketing) OR TI,AB,IF(promotion*) OR TI,AB,IF(advert*) OR TI,AB,IF(nudg*)))	76
Set#: S9	Searched for: ((TI,AB,IF(retailer?) OR TI,AB,IF(supermarket?) OR TI,AB,IF(market?) OR TI,AB,IF(shop?) OR TI,AB,IF(store?)) AND (TI,AB,IF(marketing) OR TI,AB,IF(promotion*) OR TI,AB,IF(advert*) OR TI,AB,IF(nudg*)))	1595
Set#: S10	Searched for: ((TI,AB,IF(restaurant?) OR TI,AB,IF(bistro?) OR TI,AB,IF(cafe?) OR TI,AB,IF(pub?) OR TI,AB,IF("public house?") OR TI,AB,IF(tavern?) OR TI,AB,IF(inn?)) AND (TI,AB,IF(marketing) OR TI,AB,IF(promotion*) OR TI,AB,IF(advert*) OR TI,AB,IF(nudg*)))	119

Set#: S11	Searched for: ((TI,AB,IF(cafeteria?) OR TI,AB,IF(canteen?) OR ((TI,AB,IF(dining) OR TI,AB,IF(dinner) OR TI,AB,IF(lunch)) NEAR/2 (TI,AB,IF(room?) OR TI,AB,IF(hall?)))) AND (TI,AB,IF(marketing) OR TI,AB,IF(promotion*) OR TI,AB,IF(advert*) OR TI,AB,IF(nudg*)))	61
Set#: S12	Searched for: ((TI,AB,IF(work) OR TI,AB,IF(workplace?) OR TI,AB,IF(school?) OR TI,AB,IF(college?)) AND (TI,AB,IF(marketing) OR TI,AB,IF(promotion*) OR TI,AB,IF(advert*) OR TI,AB,IF(nudg*)))	6651
Set#: S13	Searched for: TI,AB,IF("systematic review") OR TI,AB,IF(metaanalysis) OR TI,AB,IF("scoping review") OR TI,AB,IF("evidence review") OR TI,AB,IF("evidence map*") OR TI,AB,IF("evidence synthesis") OR TI(review)	60935
Set#:S14	(S1 OR S2 OR S3 OR S4 OR S5 OR S6 OR S7 OR S8 OR S9 OR S10 OR S11 OR S12) and S14	325
