# Supplementary material for: Comparing the impact and mechanistic pathways of micro-environmental interventions targeting healthier vs. more environmentally sustainable food options: an overview of reviews
Source: BMC Med. 2025 Oct 24;23:586. doi: 10.1186/s12916-025-04381-8 (PMC12553259; doi:10.1186/s12916-025-04381-8)
Supplement: Supplementary file 2 — Supplementary Material 2. Review selection & data extraction. [file 12916_2025_4381_MOESM2_ESM.pdf]

APPENDIX B. Review selection & data extraction

We included reviews with similar eligibility criteria, but assessed overlap of primary studies so that each primary study would only be included once [Pollock M, Fernandes RM, Newton AS, et al. A decision tool to help researchers make decisions about including systematic reviews in overviews of reviews of healthcare interventions. *Systematic Reviews*. 2019;8(1):29]. We reviewed abstracts in languages other than English using Google Translate. If deemed potentially eligible, we reviewed full-texts with speakers of these languages (German: independent, duplicate review; Spanish, Portuguese, French: two reviewers (CJ & EB) reviewed together with translator; Mandarin, Japanese: one reviewer (CJ) reviewed together with translator). Six articles where access was restricted were single reviewed due to copyright reasons.

We searched for full-text publications of potentially relevant conference abstracts and protocols, and if none were found, contacted authors via email. Authors of two papers could not be contacted due to missing contact details. Of seven contacted authors, four responded, three providing the full-text paper and one author advising that the study in question had never been published.

We excluded RCTs that reported results only in pre-post format within the intervention group instead of comparing results against the control group. Missing risk of bias assessments of primary studies were not re-assessed.

Deviations from the protocol

We had some deviations from the pre-registered protocol. We added an extra TIPPME category in the extraction sheet for multi-component interventions and excluded interventions aimed only at water and juice. We combined selection and purchase outcomes in analyses as not all reviews differentiated between selection with purchasing and selection without purchasing. Only a subsample of 20% of forward and backward citations at each stage were screened and extracted in duplicate due to resource limitations. Due to the substantial number of studies found, we decided not to conduct additional grey literature searches. We report results according to the PRIOR statement [Gates M, Gates A, Pieper D, et al. Reporting guideline for overviews of reviews of healthcare interventions: development of the PRIOR statement. *BMJ*. 2022;378] instead of the PRISMA statement [Page MJ, McKenzie JE, Bossuyt PM, et al. The PRISMA 2020 statement: an updated guideline for reporting systematic reviews. *BMJ*. 2021;372:n71] as it is specific to overviews of reviews. Apart from labelling interventions, we were unable to consistently apply our ex-ante defined inclusion/exclusion criteria for information interventions: *"Information interventions that only make use of a message that is expected to be visually salient (i.e. clearly seen on the typical path through the environment) and target one specific behaviour change"* (Table 2) because the description of information interventions was limited in many reviews. Instead, we decided inclusion on a case-by-case basis. Examples of which interventions we decided to exclude or include are contained in Appendix C.
