# Supplementary material for: Comparing the impact and mechanistic pathways of micro-environmental interventions targeting healthier vs. more environmentally sustainable food options: an overview of reviews
Source: BMC Med. 2025 Oct 24;23:586. doi: 10.1186/s12916-025-04381-8 (PMC12553259; doi:10.1186/s12916-025-04381-8)
Supplement: Supplementary file 3 — Supplementary Material 3. Examples of information interventions. [file 12916_2025_4381_MOESM3_ESM.pdf]

APPENDIX C. Examples of information interventions

Review	Study	Intervention description	Decision
**Atanasova_2022**	Anzman-Frasca 2018	"Placemats featuring two healthy kids' meals"	Include
**Cameron**	Huang 2006	"Intervention group: fully automated advice recommending specific switches from selected products higher in saturated fat to alternate similar products lower in saturated fat. Controls received general nonspecific advice about how to eat a diet lower in saturated fat."	Exclude
**Golding_2022**	Payne 2015	"Social norms messages placed in grocery carts: ‘in this store, most people choose at least X produce items’, graphics of most popular F&V, a smiley face & list of top 10 F&V purchased in that store"	Include
	Payne 2016	"10 large green arrows on shop floor; placed to direct shoppers’ attention to F&V section, e.g., ‘Follow green arrow for healthy [heart, weight]’"	Include
	Gamburzew 2016	"Combined strategies to target products (inexpensive, good nutritional food): (1) shelf labels, (2) posters and leaflets (3) taste testing booth and leaflets focused on canned fish, pulses and eggs"	Exclude

	Vlaeminck 2014	"Varied labels by food items (apples, tomatoes & protein items) to present details of environmental impact. Standard label (ctrl n = 50), treatment least label, information not immediately coherent (n = 50), treatment most label, information presented clearly (n = 50)" [summary label combined with specific label]	Exclude
**Harbers**	Allan 2015	"Signs visually arranged snacks and drinks from least caloric to most caloric, with arrows indicating their location in store."	Exclude
**Metcalfe**	Cohen 2015	"− Using attractive bowls or baskets − Signage and images promoting fruits and vegetables − Changing fruit and vegetable placement"	Include
	Hanks 2016	"Treatment 1: Branded vegetable characters featured on vinyl promotional banners"	Exclude
	Schwartz 2007	"− Verbal prompts promoting healthy items"	Exclude
**Stiles**	Folta 2006	"Test whether broadcasting messages over school PAk system promoting bean dishes at school lunch will increase choice of bean dishes by students"	Include
