# Supplementary material for: Comparing the impact and mechanistic pathways of micro-environmental interventions targeting healthier vs. more environmentally sustainable food options: an overview of reviews
Source: BMC Med. 2025 Oct 24;23:586. doi: 10.1186/s12916-025-04381-8 (PMC12553259; doi:10.1186/s12916-025-04381-8)
Supplement: Supplementary file 4 — Supplementary Material 4. Detailed overview of identified studies. [file 12916_2025_4381_MOESM4_ESM.pdf]

APPENDIX D. Detailed overview of identified studies

Availability

Five reviews [57,66,70,72,74] included eight interventions that evaluated the effect of a greater number of healthier options or increasing the ratio of healthier vs less healthy options on dietary behaviours.

Health

A Cochrane review of availability interventions, rated as "high" confidence based on the AMSTAR2 assessment [53], included meta-analyses of the effect of availability interventions on food selection and consumption [66]. We included two out of three studies from the selection meta-analysis, which found a large effect size of lower availability reducing selection, albeit with low certainty [66]. We included all three availability interventions from the meta-analysis of consumption outcomes, which found, with substantial uncertainty, that such interventions moderately reduce consumption [66].

Only single studies from other reviews were included, as they did not specifically aim to review availability interventions, showing mixed results. One intervention found positive effects on low-fat entrée selection after increasing the frequency of such options [72], whilst another intervention found no effect on healthier milk selection and consumption after increasing the ratio of white milk (healthy option) to chocolate milk (unhealthy option) [70]. In a virtual fast-food restaurant, increasing the availability of low-calorie meals reduced calories of hypothetical purchases [57]. One study increasing healthy options in vending machines found no effects on favourable foods purchased [74]. Two of these reviews were rated "low" [70,74] and two "critically low" [57,72].

Sustainability

No availability studies were identified relating to sustainability interventions.

Position

<u>Menus</u>

Health

We included most menu placement studies from one review (n=7/10 (70%)), which narratively summarised that the majority of such interventions showed positive effects, although effect sizes varied from no to moderate effects [63]. The interventions differed in their operationalisation. Two studies found that positioning a healthy option to the left instead of the right of an unhealthy option on an online menu increased hypothetical healthy food selections. Mixed effects were found for placing healthier items higher up a menu list, with two studies showing this was effective for online but not physical menus, while another found increased healthier hypothetical selections with altered physical menus. A third and fourth study found no evidence of impact on hypothetical selections or actual selections using online menus (although in these studies target

options were placed at both the top and bottom of menus), with one of these studies also finding no effect of top right placement. The review hypothesised that such interventions may be more effective when not all options are immediately shown (e.g. some are on the next page or require scrolling down).

One other review included a study in a sandwich restaurant: positioning listings for less healthy sandwiches inside an envelope or on the next page whilst positioning healthy sandwiches on a featured menu increased low-calorie sandwich orders (but more calories were bought overall, from other menu items) [79].

Sustainability

Placing meat options on a board further away instead of on the food menu reduced the odds of hypothetical meat selection [80]. Another study found that placing the vegetarian option first on a restaurant menu whilst noting that guests could request a meat option reduced meat meal purchases [82].

Proximity

Health

One Cochrane review targeted proximity interventions: we included 12 of the 15 (80%) interventions in the meta-analysis for consumption outcomes, which found that decreased proximity reduces consumption [66]. Two further studies included in the review but excluded from the meta-analysis also found that increasing the distance between a food item and participants reduces consumption [66]. Only two studies investigated selection, finding that positioning unhealthy snacks further away from participants reduced energy selection [66].

Other reviews included single proximity studies eligible for our review. In a laboratory study, positioning healthy snacks closer than unhealthy snacks increased their consumption [57]. In school cafeterias, needing to ask for chocolate milk instead of it being placed on stalls increased white milk selection but not consumption among students [70]. Positioning healthy options first in a buffet line increased selections in two studies, for protein bars in the dessert line of a food pantry [68], and fruit at the beginning of a breakfast buffet [79].

Sustainability

Only a single study from one review investigated a proximity intervention and sustainability outcomes, and this was not the primary intervention target: placing salad components separately at the start of a buffet instead of mixed together at the end did not affect meatball selection [80].

Defaults

Health

We included three of seven default studies (43%) from one review, which from its narrative synthesis concluded that whilst defaults appear to encourage healthier food selections, the effects on consumption are less clear [63]. Specifically, in a laboratory and a field study, a healthy default menu resulted in parents selecting healthier items for their children, whilst in simulated dining halls, a healthier default lunch option increased healthier selections but not consumption [63].

Sustainability

We included most of the default studies from another review (7/9, 78%) [82] (three of which were also identified in another review but not categorised as default interventions there [80], and one of which we classified as *menu placement* rather than default). Albeit not conducting meta-analysis, the authors concluded that defaults reduce meat consumption and that included studies were of relatively good quality [82].

Increased visibility

Health

We included one study from a review focused on positioning interventions, which found that moving healthier products to prominent positions in food stores did not impact their sales [75]. In contrast, another study from a nudging review suggested increasing visibility of fruit and vegetables in-store resulted in their increased purchases [64].

Sustainability

No studies were identified investigating the impact of increased visibility of more sustainable foods.

Presentation

No reviews specifically targeted presentation interventions.

Health

Two studies from one review investigated menu design: adding a healthy food image cue or an overweight face to a menu resulted in healthier hypothetical food choices compared to neutral cue or no image controls [63].

In terms of food shape, presenting whole wheat bread in fun shapes increased their consumption among primary school students [68]. Another study found that offering sliced versus whole apples led to increased apple sales [71]. Offering a bagel in quarters rather than as a single piece reduced consumption [65].

Other studies focused on packaging. One study found that leaving protein bars in their original packaging increased their selection as opposed to offering them in plastic bags [68]. Two interventions found that adding unfamiliar and familiar characters to food packaging increased

children's purchase requests for fruit compared to candy [65], while another study found providing children with fruit and vegetables in packages with cartoon characters did not increase their consumption [59]. In contrast, however, a fourth study found adding a popular character to foods did not increase hypothetical selection of healthier options [67].

Sustainability

One review found that changing meal presentation, such as displaying a pork roast with a pig's head vs. without the head or increasing the appeal of meat-free choices, reduces hypothetical meat meal choices, with three of four interventions finding a positive effect [80]. In another review, however, three interventions showing animal photos or using photos of low-processed meat and vegetables found no effect on intended meat consumption [81].

Functionality

Health

Two interventions from one study resulted in positive effects: a spoon with a hole and changing from bottled fish sauce to a bowl with fish sauce reduced fish sauce consumption by 0.58g and 0.25g respectively [68]. In contrast, presenting four small servings separated onto a tray and three lunch boxes rather than serving one large portion did not affect consumption [65].

Sustainability

We found no functionality interventions aimed at sustainability.

Size

<u>Tableware</u>

Health

A Cochrane review with meta-analyses of tableware interventions found that smaller tableware reduced consumption (n=12) and selection (n=7) respectively [65]. Another intervention not included in the meta-analysis also found that smaller size reduced selection [65]. A different meta-analysis of the impact of tableware size interventions similarly found that smaller tableware reduces portion size selections and consumption (n=7/8 (88%) of selection and n=17/21 (81%) of consumption comparisons were included in this review) [77], with considerable overlap with the Cochrane review [65]. Two other studies not included in the selection meta-analysis either did not find an effect or found a smaller bowl increased portion size selected [77].

Sustainability

No interventions were identified here for sustainability.

<u>Portions</u>

Health

A Cochrane meta-analysis of 58 portion size interventions found smaller portion sizes reduced consumption [65]. We included three of five interventions from the selection metaanalysis, which found that smaller portions reduced energy selected [65]. Of eight more portion size interventions not incorporated into the meta-analysis, five found smaller portions reduced consumption, two found no effects and one found a negative effect [65].

Two studies from other reviews suggested that offering a reduced portion size resulted in switching to smaller meals for around 10% of purchases (with no evidence of compensatory purchasing) [55], and that reducing rice portion sizes decreased consumption [77].

Sustainability

One review using qualitative comparative analysis found that reducing meat portions lowers meat consumption [80], although two of three interventions overlapped with another review (reported under health, as investigating overall energy intake instead of meat intake specifically) [65]. Stocking a smaller portion size of sausages in a store reduced meat sales [82].

Packages

Health

A meta-analysis including 10 comparisons, of which we included 9 in our review (although we characterised one intervention as functionality rather than size), found that smaller packages reduced consumption [65]. Another intervention recorded no effect of package size on consumption. One package intervention found smaller packages reduce selection [65].

Another intervention from a different review found no effect of peanut butter jar size on the amount of peanut butter spread onto bread [59].

Sustainability

No interventions were identified here for sustainability.

Individual units

Health

Only one review [65] included interventions investigating the effect of individual unit size: three comparisons found no effect on food selection; in terms of consumption, two comparisons found that smaller individual unit size reduced consumption whilst five did not find an effect.

Sustainability

No interventions were identified here for sustainability.

Information

Labels

Health

Calling out healthy options: A green tick label resulted in increased nutritional quality of hypothetical food selections in one study [73], however, in another, a healthy choice tick did not find a difference in intended consumption of foods [51]. Similarly, in three other studies, healthy choices logos were not found to impact on hypothetical or actual food purchases or consumption [51,56,64].

Labelling all items: A health star label was found to be effective in reducing hypothetical sugary drink choices [62] but did not affect purchases of sugary drinks [56]. Furthermore, a healthiness star rating label did not affect the healthiness of purchases in university vending machines [78]. Two studies investigating 5-colour nutrition labels found positive and no effects on nutritional quality of hypothetical food purchases [56,73], while two nutriscore label interventions found no difference in intended purchases [69,76]. In contrast, a NuVal label resulted in significant improvements in NuVal scores of hypothetical purchases [58].

Sustainability

Two labels calling out the more sustainable options were effective in increasing more sustainable food purchases for women but not men [83]. A leaf symbol did not have an effect on hypothetical food choices [80].

Social norms

Health

One review included two social norms interventions: an intervention implementing social norm messages on shopping carts recorded increased purchases of fruit and vegetables, while banners with a social norms message did not increase low-fat cheese choices in a supermarket [61].

Sustainability

A review from which we included most of the prompt or social norm studies (5/7; 71%) found that two of five dynamic norm interventions increased vegetarian sales, with two finding no effect and one intervention discouraging vegetarian orders [83]. One static norm intervention showed no difference to control, while a label implying a social norm (*"Student choice"*) increased vegetarian purchases in women but not men [83]. Three injunctive social norm interventions from another review found no effect on purchases of fish patties in a factorial RCT [13].

Changed food dish descriptions

Health

Only one study from one review was included, which found that changing names of healthy dishes to use more descriptive language did not impact intended food choices in an assisted living centre [63].

Sustainability

One review included three interventions changing the description of dishes, for example adding "Chef's recommendation" to dishes or using the words "cow" and "pig" instead of "beef" and "pork", none of them affecting intended food selection [80].

Other

Health

Using green arrows to signpost store customers towards healthier choices was associated with increased healthy purchases [61]. Placemats promoting healthy kids meals in a restaurant were also found to increase purchases and intake of healthier meals [57].

Sustainability

Advertising bean dishes via school announcement systems had no effect on overall selection of these dishes [83].

Multicomponent

Health

Two multicomponent interventions found positive effects on fruit selection: one intervention combined positioning, presentation, availability and information, and the other presentation, information and positioning intervention elements [71]. The former also found increases in fruit consumption, but the latter found no differences in fruit intake [71]. Two interventions combined availability, positioning and information, one identified in each of two reviews [60,71], both finding reduced energy purchases or intake.

Sustainability

No interventions were identified here for sustainability.
