# Supplementary material for: Comparing the impact and mechanistic pathways of micro-environmental interventions targeting healthier vs. more environmentally sustainable food options: an overview of reviews
Source: BMC Med. 2025 Oct 24;23:586. doi: 10.1186/s12916-025-04381-8 (PMC12553259; doi:10.1186/s12916-025-04381-8)
Supplement: Supplementary file 5 — Supplementary Material 5. Mediators, moderators and cross-cultural factors. [file 12916_2025_4381_MOESM5_ESM.pdf]

APPENDIX E. Mediators, moderators and cross-cultural factors.

Intervention characteristics

Size: We included most, but not all, studies from a Cochrane review which identified that the impact of size on consumption was greater for more energy-dense or less healthy items [65]. However, this was not true for selection outcomes [65]. Effects on consumption were also larger if there were no other, non-manipulated items available and intake was ad-libitum. There was no evidence of a moderating effect based on whether the portion or package was manipulated for selection, or portion, package, individual unit, or tableware size was manipulated for consumption [65]. There was also no evidence of the absolute or relative size difference between two options affecting consumption or selection [65].

Proximity: We included most of the proximity interventions included from a Cochrane review [66], which found two effect modifiers of the effect of increasing the proximity of a product on consumption: effects were larger if there were no other, non-manipulated items available and with decreasing proximity of a comparator product. Product healthiness and duration of exposure however did not modify the effect.

Defaults: A sustainability-focused review of default interventions from which we included the majority of studies reported an ex-post exploration of potential mediators, and hypothesised that endorsement (e.g., perceiving the default as the advised or expected choice) and effort are some of the underlying mechanisms [82]. The same review explored potential moderators and we included seven of the twelve studies that this exploration was based on [82]. They identified the intrusiveness of the default, how the alternative to the default is presented or can be recognised, and the setting's objective as potential moderators.

Menu design: One health-focused study in one review found no mediating effect of visual attention for the link between a menu presentation intervention and food selection [63].

Labels & social norms: There was no additional benefit of combining a student choice and a sustainability label for vegetarian purchases [83].

Participant characteristics

Socio-economic position (SEP): One review from which we included most of the proximity interventions found the effect of decreasing the proximity of a product on consumption was larger in low socioeconomic deprivation contexts [66] but no difference was found for size interventions in another review [65]. One review included a study where a summary label led to higher intended sodium intake in participants with lower levels of education [51]. Another study from a different review found that the impact of health labels (green ticks and 5-colour nutrition labels) was smaller in those with lower income [73]. However, education and income were not found to be moderating the effect of a nutriscore label in another study from another review [76]. Another review found that a positioning intervention aimed at health had a larger impact for participants who were on a food assistance program [64].

Gender: A Cochrane review of size interventions found no evidence of differential effects by gender on food consumption or selection [65]. Gender did not moderate the effect of proximity interventions on consumption in another Cochrane review [66]. Two labelling and one social norm intervention increased sustainable purchases for women but not men [83].

Age: A Cochrane review found that effects of larger sizes increasing consumption were larger in older participants and for selection, there was an effect for adults but not children [65].

Previous experience: One intervention from a review of size interventions reported that the effect of a sugar spoon size intervention for tea was stronger in participants who had a weaker habit of adding a specific amount of sugar to their tea [77]. Additionally, a social norm intervention found that participants who had stronger campus identification and had made a purchase in the previous year were more likely to purchase a vegetarian meal [83]. One size intervention found that a larger amount of food was self-served if participants ate with friends as opposed to strangers [77].

Other: A Cochrane review reported no differences in the impact of size interventions on food consumption based on BMI, dietary restraint, dietary disinhibition, or hunger [65].[1] In one study included from another review [76], hunger and mood were not found to be moderating the effect of a nutriscore label. In one review, a study found no interaction between egodepletion and a social norm message for the purchase of low-fat cheese [61].

No cross-cultural factors were mentioned in any review regarding any of the included studies.

[1] This review assessed >30 variables in meta-regression analyses, many of which are not explicitly interpreted in the review beyond their statistical data. We therefore only report here those covariates that are explicitly mentioned in the review.
